# Prognostic Significance and Associations of Neural Network–Derived Electrocardiographic Features

**DOI:** 10.1161/CIRCOUTCOMES.123.010602

**Published:** 2024-11-14

**Authors:** Arunashis Sau, Antônio H. Ribeiro, Kathryn A. McGurk, Libor Pastika, Nikesh Bajaj, Mehak Gurnani, Ewa Sieliwonczyk, Konstantinos Patlatzoglou, Maddalena Ardissino, Jun Yu Chen, Huiyi Wu, Xili Shi, Katerina Hnatkova, Sean L. Zheng, Annie Britton, Martin Shipley, Irena Andršová, Tomáš Novotný, Ester C. Sabino, Luana Giatti, Sandhi M. Barreto, Jonathan W. Waks, Daniel B. Kramer, Danilo Mandic, Nicholas S. Peters, Declan P. O’Regan, Marek Malik, James S. Ware, Antonio Luiz P. Ribeiro, Fu Siong Ng

**Affiliations:** National Heart and Lung Institute (A.S., K.A.M., L.P., N.B., M.G., E. Sieliwonczyk, K.P., M.A., J.Y.C., H.W., X.S., K.H., S.Z., D.B.K., N.S.P., M.M., J.S.W., F.S.N.), Imperial College London, United Kingdom.; Medical Research Council Laboratory of Medical Sciences (K.A.M., E. Sieliwonczyk, D.P.O., J.S.W.), Imperial College London, United Kingdom.; Department of Electrical and Electronic Engineering (D.M.), Imperial College London, United Kingdom.; Department of Cardiology, Imperial College Healthcare National Health Service Trust, London, United Kingdom (A.S., N.S.P., F.S.N.).; Department of Information Technology, Uppsala University, Sweden (A.H.R.).; Faculty of Medicine and Health Sciences, Center for Medical Genetics, University of Antwerp and Antwerp University Hospital, Antwerp, Belgium (E. Sieliwonczyk).; Research Department of Epidemiology and Public Health, University College London, United Kingdom (A.B., M.S.).; Department of Internal Medicine and Cardiology, University Hospital Brno and Masaryk University, Czech Republic (I.A., T.N., M.M.).; Department of Infectious Diseases, School of Medicine and Institute of Tropical Medicine, University of São Paulo, Brazil (E. Sabino).; Department of Preventive Medicine, School of Medicine, and Hospital das Clínicas/Empresa Brasileira de Serviços Hospitalares (L.G., S.M.B.), Universidade Federal de Minas Gerais, Belo Horizonte, Brazil.; Department of Internal Medicine, Faculdade de Medicina, and Telehealth Center and Cardiology Service, Hospital das Clínicas (A.L.P.R.), Universidade Federal de Minas Gerais, Belo Horizonte, Brazil.; Harvard-Thorndike Electrophysiology Institute (J.W.W.), Beth Israel Deaconess Medical Center, Harvard Medical School, Boston, MA.; Richard A. and Susan F. Smith Center for Outcomes Research in Cardiology (D.B.K.), Beth Israel Deaconess Medical Center, Harvard Medical School, Boston, MA.; Department of Cardiology, Royal Brompton and Harefield Hospitals, Guy’s and St. Thomas’ NHS Foundation Trust, London, United Kingdom (J.S.W.).; Department of Cardiology, Chelsea and Westminster Hospital NHS Foundation Trust, London, United Kingdom (F.S.N.).

**Keywords:** cardiovascular diseases, electrocardiography, neural networks, computer, supervised machine learning, unsupervised machine learning

## Abstract

**BACKGROUND:**

Subtle, prognostically important ECG features may not be apparent to physicians. In the course of supervised machine learning, thousands of ECG features are identified. These are not limited to conventional ECG parameters and morphology. We aimed to investigate whether neural network–derived ECG features could be used to predict future cardiovascular disease and mortality and have phenotypic and genotypic associations.

**METHODS:**

We extracted 5120 neural network–derived ECG features from an artificial intelligence–enabled ECG model trained for 6 simple diagnoses and applied unsupervised machine learning to identify 3 phenogroups. Using the identified phenogroups, we externally validated our findings in 5 diverse cohorts from the United States, Brazil, and the United Kingdom. Data were collected between 2000 and 2023.

**RESULTS:**

In total, 1 808 584 patients were included in this study. In the derivation cohort, the 3 phenogroups had significantly different mortality profiles. After adjusting for known covariates, phenogroup B had a 20% increase in long-term mortality compared with phenogroup A (hazard ratio, 1.20 [95% CI, 1.17–1.23]; *P*<0.0001; phenogroup A mortality, 2.2%; phenogroup B mortality, 6.1%). In univariate analyses, we found phenogroup B had a significantly greater risk of mortality in all cohorts (log-rank *P*<0.01 in all 5 cohorts). Phenome-wide association study showed phenogroup B had a higher rate of future atrial fibrillation (odds ratio, 2.89; *P*<0.00001), ventricular tachycardia (odds ratio, 2.00; *P*<0.00001), ischemic heart disease (odds ratio, 1.44; *P*<0.00001), and cardiomyopathy (odds ratio, 2.04; *P*<0.00001). A single-trait genome-wide association study yielded 4 loci. *SCN10A*, *SCN5A*, and *CAV1* have roles in cardiac conduction and arrhythmia. *ARHGAP24* does not have a clear cardiac role and may be a novel target.

**CONCLUSIONS:**

Neural network–derived ECG features can be used to predict all-cause mortality and future cardiovascular diseases. We have identified biologically plausible and novel phenotypic and genotypic associations that describe mechanisms for the increased risk identified.

**WHAT IS KNOWN**

- Conventional ECG parameters, such as QRS duration, are associated with prognosis.
- During supervised deep learning model training, thousands of neural network–derived ECG features are identified.

**WHAT THE STUDY ADDS**

- Phenogroups derived using neural network–derived ECG features can predict future cardiovascular disease and death.
- Explainability analyses demonstrate these phenogroups have important phenotypic and genotypic associations.

**See Editorial by Sangha and Khera**

The ECG is a widely used investigation for the assessment of cardiovascular disease. The ECG captures information relating to a wide range of cardiac pathology, from cardiomyopathy and channelopathies to conduction system disease. Several specific ECG features have been associated with adverse long-term prognosis, including left bundle branch block,^1^ prolongation of QRS duration,^2^ and ECG changes consistent with left ventricular hypertrophy.^3^ However, these crude ECG features are based on physician interpretation or ECG measurements, and there may be other ECG features, not easily discernible by humans, that may be as clinically and prognostically meaningful.

Recently, there has been a rapid increase in applications of artificial intelligence to the analysis of the ECG.^4–6^ Supervised machine learning (ML) is the most common form of ML applied to ECGs, where a model is trained to predict a label, for example, future atrial fibrillation or impaired left ventricular function.^5,6^ Artificial intelligence–enabled electrocardiography (AI-ECG) models often use a convolutional neural network (CNN) architecture and have generally performed well.^4–6^ Although the classification task is often specific and singular, the CNN identifies thousands of ECG features over the course of model training, which are not limited to conventional ECG parameters and morphology, and applies these features to make the label prediction.^7^ These novel neural network (NN)–derived ECG features have not been extensively studied. There is the potential that they may be universal, transferable, and applicable to a range of clinically useful tasks.

We hypothesized that the ECG features learned by an NN may have implications beyond the original task for which it was trained and may have important clinical, phenotypic, and genotypic associations. In a transfer learning–based approach, we applied an existing AI-ECG model trained to classify 6 common ECG diagnoses relating to rhythm and conduction disease.^8^ We extracted the NN-derived ECG features from the penultimate layer of the CNN and applied unsupervised ML to identify clinically distinct phenogroups with prognostic significance. Given the critical role of external validation,^9^ we validated our analysis in 5 external data sets across 2 continents with diverse ancestral backgrounds. Uniquely, our model was evaluated across diverse cohorts, including volunteers, primary care patients, and patients with established cardiomyopathy. Finally, we describe novel biological insights underlying the associations of the ECG phenogroups with survival by evaluating their associations with a wide range of phenotypes and genetic variants.

## Methods

### Data Availability

The São Paulo-Minas Gerais Tropical Medicine Research Center (SaMi-Trop) cohort was made openly available (https://doi.org/10.5281/zenodo.4905618). The Clinical Outcomes in Digital Electrocardiography (CODE)-15% cohort was also made openly available (https://doi.org/10.5281/zenodo.4916206). Restrictions were applied to additional clinical information on the CODE-15% and SaMi-Trop cohorts; to the full CODE cohort, the ELSA-Brasil (Longitudinal Study of Adult Health) cohort was applied. UK Biobank (UKB) data are available upon application (http://www.ukbiobank.ac.uk/). Whitehall II data are available upon application (https://www.ucl.ac.uk/psychiatry/research/mental-health-older-people/whitehall-ii). The Beth Israel Deaconess Medical Center (BIDMC) data set is restricted due to ethical limitations. Researchers affiliated to educational or research institutions may make requests to access the data sets. Requests should be made to the corresponding author of this article. They will be forwarded to the relevant steering committee.

### Code Availability

The code and model weights of CODE-CNN are available at https://github.com/antonior92/automatic-ecg-diagnosis. The remaining programming code will be made available upon reasonable request to the corresponding author.

### Ethical Approval

This study complies with all relevant ethical regulations, including institutional review board approval; full details are provided in Supplemental Methods.

### ECG Data Sets

We studied 6 cohorts; briefly, the CODE cohort is a Brazilian database of ECGs recorded in primary care.^10^ The Whitehall II cohort consists of British civil servants.^11^ The UKB is a longitudinal study of volunteers.^12^ The ELSA-Brasil cohort consists of Brazilian public servants.^13^ SaMi-Trop is a cohort of patients with chronic Chagas cardiomyopathy,^14^ and finally the BIDMC cohort is a secondary care data set that comprised routinely collected data from Boston, MA. Full details are provided in Supplemental Methods.

### ECG Preprocessing

Twelve-lead ECGs were preprocessed by removing the baseline drift and resampling to 400 Hz. Zero padding resulted in a signal with 4096 samples for each lead for a 10-second recording, which is used as input to the NN model. No normalization was performed.

### NN-Derived ECG Features From CODE Data Set

The CODE data set was previously used to train a CNN to detect 6 common ECG abnormalities (first-degree atrioventricular block, right bundle branch block, left bundle branch block, sinus bradycardia, atrial fibrillation, and sinus tachycardia), as previously described.^8^ This model is referred to as CODE-CNN. The Keras framework with a TensorFlow backend was used for NN training and inference.^15,16^

**Figure 1.**
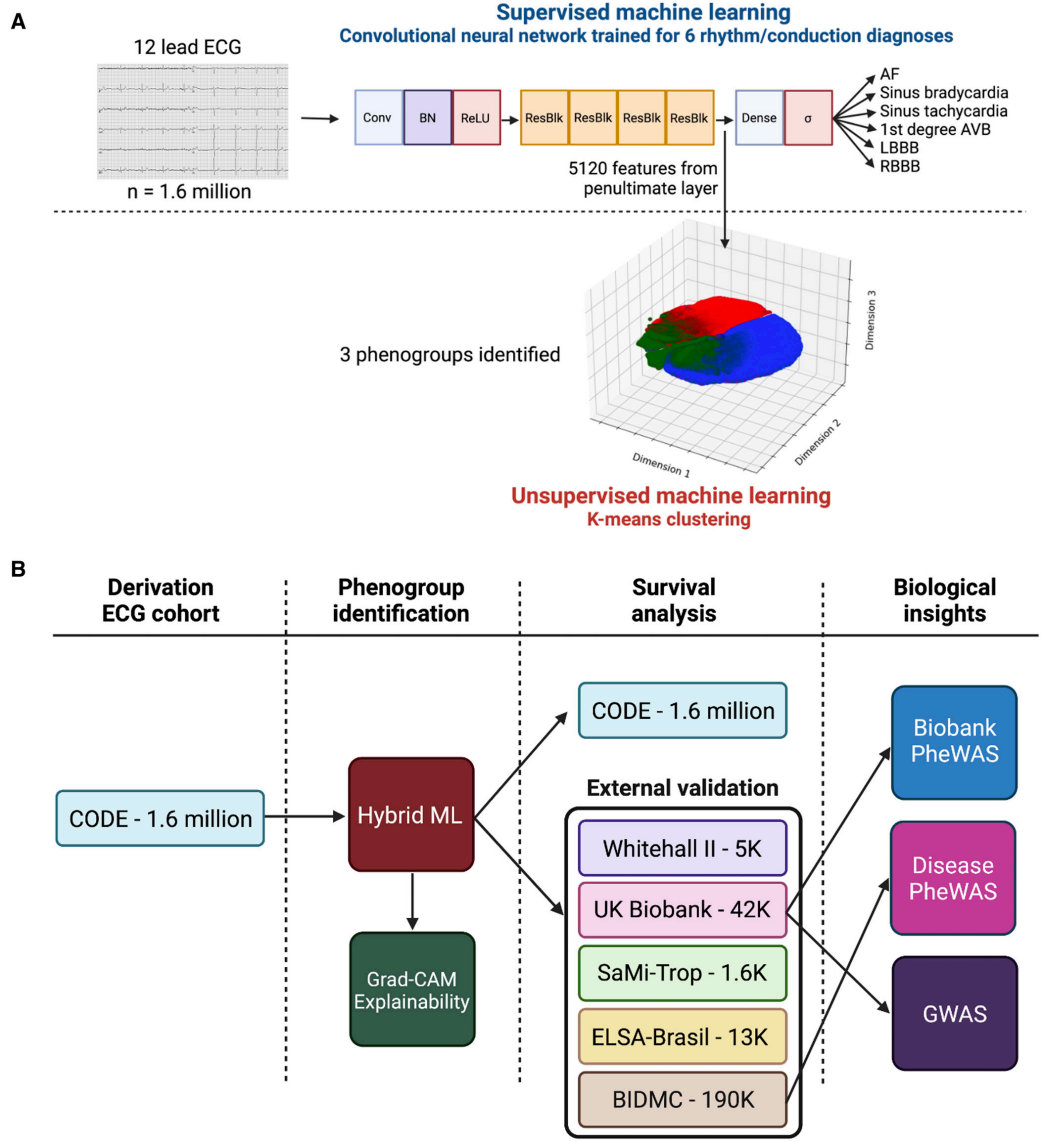
**Data analysis pipeline. A**, Hybrid machine learning approach with a combination of supervised and unsupervised machine learning to use neural network–derived ECG features to identify phenogroups from the 12-lead ECG. Three-dimensional figure shows a dimensionally reduced representation of the phenogroups. **B**, Flow of data and analyses performed in this study. AF indicates atrial fibrillation; AVB, atrioventricular block; BIDMC, Beth Israel Deaconess Medical Center; BN, batch normalization; Conv, convolutional layer; CODE, Clinical Outcomes in Digital Electrocardiography; ELSA-Brasil, Brazilian Longitudinal Study of Adult Health; Grad-CAM, gradient-weighted class activation mapping; GWAS, genome-wide association study; LBBB, left bundle branch block; ML, machine learning; PheWAS, phenome-wide association study; ReLu, rectified linear unit; ResBlk, residual block; RBBB, right bundle branch block; and SaMi-Trop, São Paulo-Minas Gerais Tropical Medicine Research Center.

Transfer learning can be used to repurpose a ML model trained for one task to another. We hypothesized the ECG features learned by the CODE-CNN could be used to cluster subjects into clinically meaningful phenogroups. In a transfer learning–based approach, the final classification layer of the model was removed (Figure 1), and the output of 5120 NN-derived ECG features is used to cluster the subjects. The NN-derived ECG features are the embeddings extracted by CODE-CNN.

### Unsupervised ML for Clustering Into Phenogroups

To derive phenogroups using the 5120 features extracted using the CODE-CNN model, K-means clustering was applied in the CODE data set using all 5120 features, without any further dimensionality reduction. k=3 was selected using the elbow method (Figure S1).^17^ Full details are provided in Supplemental Methods. Cluster centroids were derived in the CODE data set, and cluster assignments for the other data sets were performed based on these fixed centroids without further fine-tuning or changing of cluster centroids. To visualize the phenogroups, principal component analysis was performed, followed by t-distributed stochastic neighbor embedding of the top 50 principal components.

### Survival Analysis in Derivation and External Validation Cohorts

Survival analysis for the 3 phenogroups was first performed for the CODE data set and then in the 5 external validation data sets (UKB, Whitehall II, ELSA-Brasil, SaMi-Trop, and BIDMC). Kaplan-Meier plots were used to display cumulative mortality. The log-rank test was used to compare survival curves. Cox proportional hazards regression modeling was used to estimate hazard ratios (HRs) for mortality while correcting for other known variables. QRS microfragmentation was included as a covariate in the Whitehall II data, and the methodology has been previously described.^18^ Further details are provided in Supplemental Methods.

### Phenome-Wide Association Study

To better understand the biology underlying our AI-ECG model and to explore the detailed phenogroup associations, we performed phenome-wide association studies (PheWAS). In the UKB, phenogroup C had limited representation and, therefore, was excluded from this analysis. We used 2 PheWAS approaches; first, we used a disease PheWAS to explore the association of the ECG phenogroup with incident diseases and treatments. Logistic regression was performed to investigate this association. The second approach, Biobank PheWAS, used the UKB that contains data from over 3000 phenotypes derived from patient measurements, surveys, and investigations. Univariate correlation was performed to investigate the association between ECG phenogroups and phenotypes as previously described.^19^ The phenogroup variables were encoded as 0 (phenogroup A) and 1 (phenogroup B). Further details are provided in Supplemental Methods.

### Genome-Wide Association Study

To identify genetic associations with the ECG phenogroups, we performed a genome-wide association study (GWAS). Subjects in phenogroup C were excluded from this analysis due to the small number of subjects, as described above for PheWAS. Using logistic regression, a binary phenogroup variable was adjusted for the following covariates: age at imaging visit, sex, height, body mass index, and the first 10 genetic principal components. Further details are provided in Supplemental Methods.

### Model Explainability

To understand the elements of the ECG contributing most significantly to phenogroup determination, we modified a commonly used technique in computer vision, gradient-weighted class activation mapping.^20^ Further details are provided in Supplemental Methods.

## Results

An overview of the analysis pipeline is provided in Figure 1.

### Derivation Cohort

Using the first recorded ECG per patient, 1 558 421 ECGs in the CODE data set were available. Patients were followed up for a mean of 3.68±1.87 years. Notably, 52 127 (3.3%) subjects died during the follow-up period. Using a transfer learning–based hybrid ML pipeline (Figure 1), 3 phenogroups were identified, and the elbow method was used to determine the number of phenogroups (further details in Methods section). Detailed subplots depicting the distributions of the clusters are shown in Figure S2 and Table S1. The demographic distribution of these 3 phenogroups is shown in Table S2. Sensitivity analysis with values of K from 2 to 9 is shown in Figure S3.

### Survival Analysis

**Figure 2.**
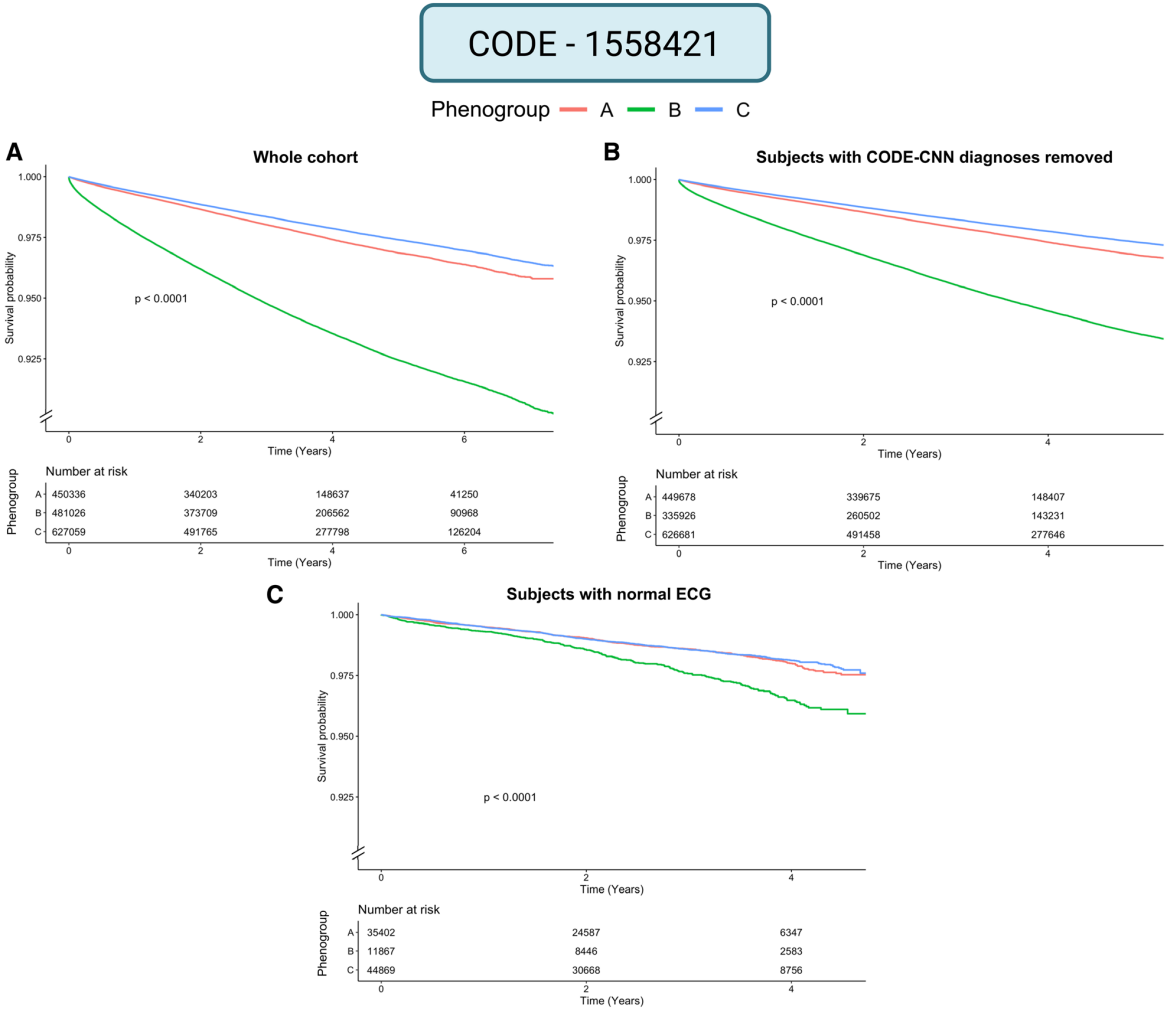
**Survival analysis in the derivation data set.** The 3 phenogroups have prognostic significance, with phenogroup B having a markedly worse prognosis. Data are shown for (**A**) the whole Clinical Outcomes in Digital Electrocardiography (CODE) cohort and (**B**) the CODE cohort with the removal of subjects with any of the following diagnoses on the ECG: first-degree atrioventricular block, right bundle branch block, left bundle branch block, sinus tachycardia, sinus bradycardia, and atrial fibrillation. **C**, Subset of the CODE cohort with normal ECGs. CODE-CNN indicates CODE-convolutional neural network.

Our survival analysis demonstrated the 3 phenogroups had different mortality profiles (Figure 2A). Phenogroup B had a 2.57-fold higher risk of mortality compared with phenogroup A, while phenogroup C had a 15% lower risk (phenogroup B versus A: HR, 2.57 [95% CI, 2.51–2.63]; *P*<0.001; phenogroup C versus A: HR, 0.85 [95% CI, 0.82–0.87]; *P*<0.001). We additionally explored how position within phenogroup B is associated with mortality risk and found subjects at the peripheries of the overall distribution but closest to phenogroup B had the highest mortality risk (Supplemental Results). The prognostic significance of the phenogroups was retained after adjusting for known covariates (age, sex, known cardiovascular comorbidities, and the 6 ECG diagnoses on which the NN was trained; Table S3). In these adjusted analyses, phenogroup B (highest mortality) had a 1.2-fold increase in long-term mortality compared with phenogroup A (HR, 1.20 [95% CI, 1.17–1.23]; *P*<0.0001; Table S3).

We then evaluated the performance of the phenogroups in the group of patients without any of the 6 diagnoses for which CODE-CNN was originally trained. Importantly, the predictive ability of the phenogroups was retained in this group (n=1 412 285; Figure 2B; Table S3). We also analyzed a subgroup of patients with physician-adjudicated normal ECGs (n=92 138). In this analysis, unadjusted phenogroup B was prognostically significant (phenogroup B versus A: HR, 1.65 [95% CI, 1.43–1.92]; *P*<0.0001; Figure 2C) but lost significance after adjustment for covariates (Table S3).

### External Validation

We then externally validated our findings in 5 diverse cohorts. There were 5066 subjects available for analysis in the Whitehall II study. For this analysis, events were censored at 5 years of follow-up. In all, 163 (3.22%) subjects died during this period. In the UKB, there were 42 386 subjects available for analysis. Patients were censored at the time of their first event, and 411 (0.97%) subjects died during the follow-up period of 3.73±1.57 years. Given the comparatively significantly lower mortality rate and the additional data available in the UKB, the primary end point for this data set was specified as major adverse cardiovascular event (definition described in Methods section), but mortality is also reported. In all, 967 (2.3%) subjects had a major adverse cardiovascular event during follow-up. The ELSA-Brasil cohort had 13 739 subjects available for analysis. The mean follow-up was 9.35±1.28 years, and 599 (4.4%) subjects died during follow-up. The SaMi-Trop cohort had 1631 subjects available for analysis. The mean follow-up period was 2.08±0.39 years, and 104 (6.38%) subjects died during the follow-up period. Lastly, the BIDMC cohort consists of a secondary care population. Notably, 188 972 subjects were available for analysis. The mean follow-up period was 5.46±5.81 years, and 34 851 (18.4%) subjects died during follow-up. The demographic distribution of these cohorts is shown in Table S2.

**Figure 3.**
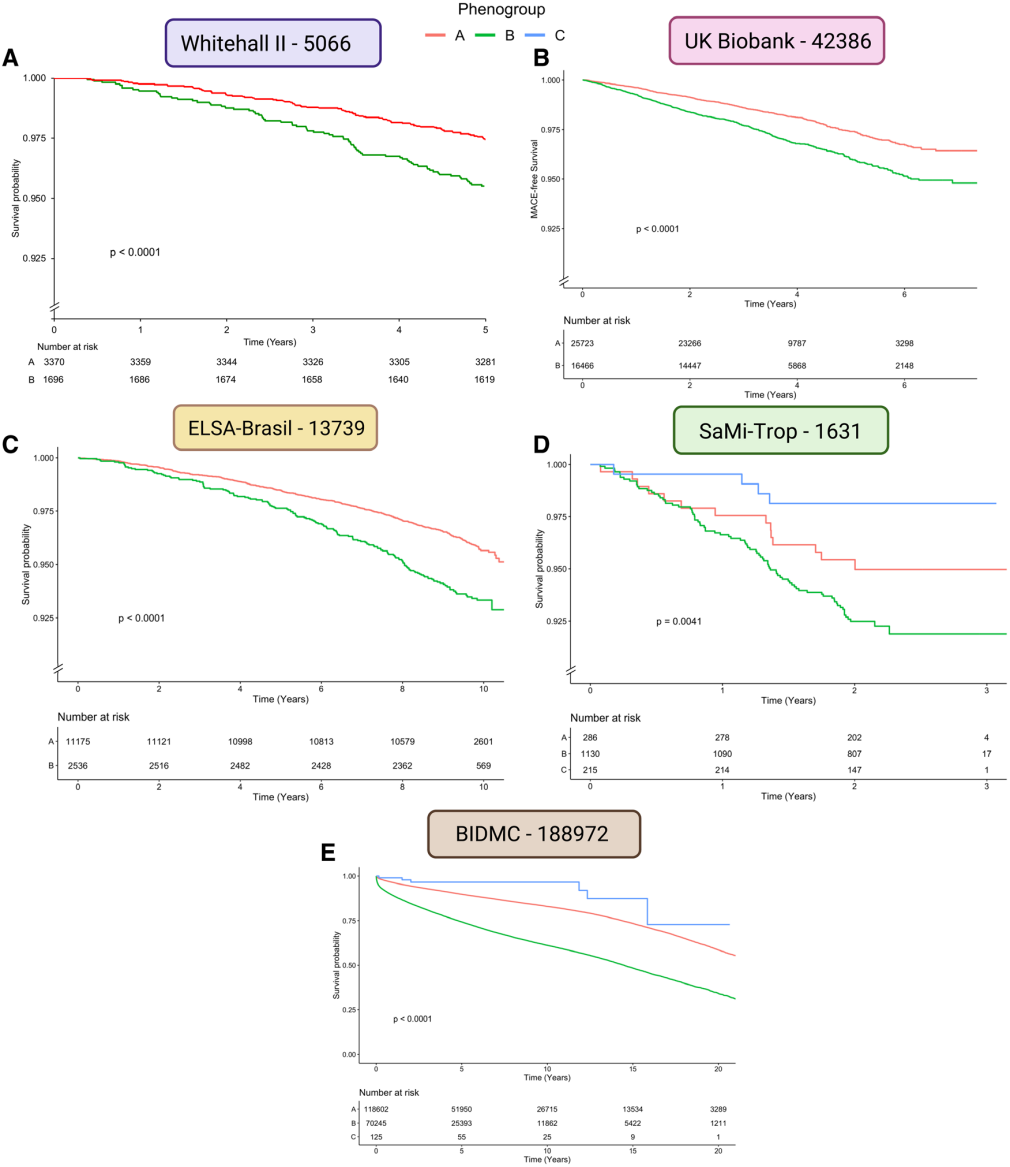
**Survival analysis in the 5 external validation data sets.** In the volunteer populations (Whitehall II, UK Biobank, and Brazilian Longitudinal Study of Adult Health (ELSA-Brasil), phenogroup C has few subjects and, therefore, is excluded. Phenogroup B has a significantly higher event rate. **A**, Whitehall II cohort and (**B**) UK Biobank, survival free of major adverse cardiovascular events is depicted. **C**, ELSA-Brasil cohort; **D**, São Paulo-Minas Gerais Tropical Medicine Research Center (SaMi-TROP) cohort; and **E**, Beth Israel Deaconess Medical Center (BIDMC) cohort.

The prognostic significance of the phenogroups was demonstrated in all of the external cohorts (Figure 3). The BIDMC secondary care population had a significantly higher mortality rate than other cohorts and accordingly had the largest absolute risk difference between phenogroups. In the volunteer cohorts (Whitehall II, UKB, and ELSA-Brasil), phenogroup C had limited representation and, therefore, was excluded from analysis. Further exploration of phenogroup C is provided in Supplemental Results. This may be due to the relatively selected population enrolled in these cohorts. Cox regression models adjusted for known covariates showed the phenogroups retained their prognostic significance in the CODE, UKB, Whitehall II, and BIDMC cohorts but not ELSA-Brasil. Importantly, this included adjustments for standard ECG parameters in the UKB, Whitehall II, and BIDMC cohorts.

To investigate the prognostic significance of the phenogroups in a more specific disease context, the SaMi-Trop cohort was analyzed. In contrast to the volunteer cohorts described above (who would be expected to largely have relatively normal ECGs), most patients in the SaMi-Trop cohort had abnormal ECGs.^21^ In this cohort of patients with chronic Chagas cardiomyopathy, the 3 phenogroups had marked prognostic significance (Figure 3C), although the Cox model adjusted for age and sex was not statistically significant (Table S3).

In BIDMC, UKB, and Whitehall II, we additionally analyzed the association of phenogroup B with cardiovascular versus noncardiovascular death. In Whitehall II, for all-cause mortality, ECG phenogroup remained a significant predictor of all-cause mortality after adjustment for age, heart rate, QTc, QRS duration, micro-QRS fragmentation, and QRS-T angle (162 events: HR, 1.61 [95% CI, 1.155–2.236]; *P*=0.005). This finding was significant for noncardiovascular mortality (114 events: HR, 1.54 [95% CI, 1.04–2.28]; *P*=0.032). Cardiovascular mortality was less common in this cohort, there was a trend toward statistical significance (48 events: HR, 1.72 [95% CI, 0.92–3.18]; *P*=0.09). Phenogroup B was associated with a higher HR for cardiovascular death compared with noncardiovascular death in both BIDMC and UKB (BIDMC cardiovascular death: HR, 3.22 [95% CI, 3.07–3.38]; *P*<0.0001; UKB: HR, 2.63 [95% CI, 1.78–3.88]; *P*<0.0001; BIDMC noncardiovascular death: HR, 2.36 [95% CI, 2.31–2.42]; *P*<0.0001; UKB: HR, 1.21 [95% CI, 0.996–1.47]; *P*=0.055; Table S4).

We went on to perform survival analysis on the subset of ECGs without any of the original rhythm/conduction diagnoses for which CODE-CNN was trained (Figure S4). Phenogroup B had statistically significant higher mortality in the UKB, Whitehall II, and BIDMC cohorts. Phenogroup B trended toward higher mortality but was nonsignificant in the comparatively smaller cohorts of SaMi-Trop (*P*=0.05) and ELSA-Brasil (*P*=0.11).

### Phenome-Wide Association Study

**Figure 4.**
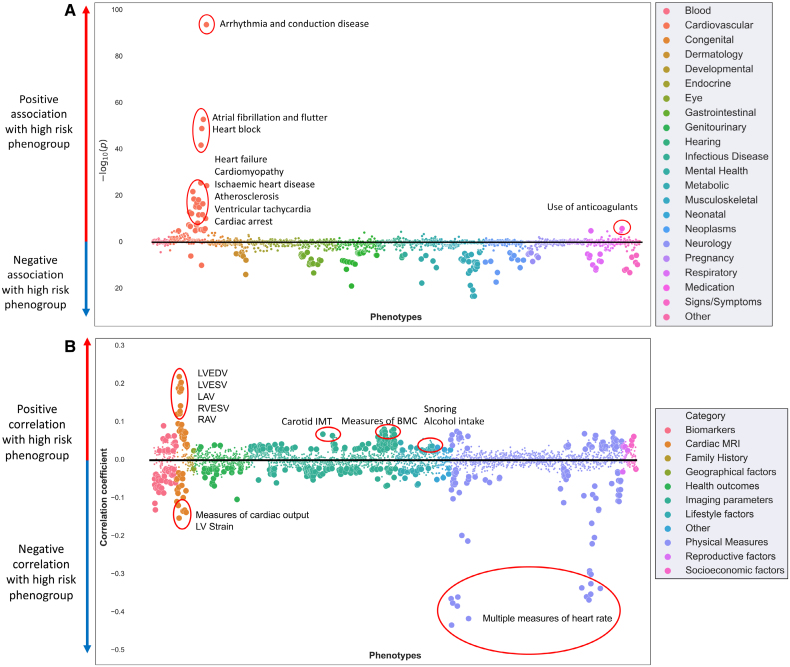
**Phenome-wide association study (PheWAS).** Manhattan plot showing negative logarithm of the univariate correlation *P* value between phenotypes for Beth Israel Deaconess Medical Center (BIDMC) disease phecodes, Disease PheWAS (**A**), and correlation coefficient for UK Biobank phenotypes, Biobank PheWAS (**B**). Small points depict associations not reaching statistical significance, while large points crossed the Bonferroni threshold for statistical significance. BMC indicates bone mineral content; IMT, intima-media thickness; LAV, left atrial volume; LV, left ventricle; LVEDV, left ventricular end-diastolic volume; LVESV, left ventricular end-systolic volume; MRI, magnetic resonance imaging; RAV, right atrial volume; RV, right ventricle; and RVESV, left ventricular end-systolic volume.

To evaluate phenotypic associations with the ECG phenogroups, we performed PheWAS using 2 approaches. First, we evaluated incident disease in the BIDMC cohort (Figure 4A). We found the high-risk phenogroup (phenogroup B) was associated with a significantly higher rate of future atrial fibrillation (odds ratio [OR], 2.89; *P*<0.00001), ischemic heart disease (OR, 1.44; *P*<0.00001), atrioventricular block (OR, 4.88; *P*<0.00001), cardiomyopathy (OR, 2.04; *P*<0.00001), ventricular tachycardia (OR, 2.00; *P*<0.00001), and cardiac arrest (OR, 6.68; *P*<0.00001). Second, we investigated phenotypes in the UKB. Figure 4B shows the Manhattan plot of the univariate correlation *P* values and correlation coefficients between ECG phenogroups and non-ECG phenotypes. In all, 512 of 3142 comparisons reached the Bonferroni threshold for significance. These included increased cardiac chamber volumes, which positively correlated with phenogroup B (higher major adverse cardiovascular event rate). Left ventricular ejection fraction, right ventricular ejection fraction, and measures of left ventricular strain were negatively correlated. The findings were similar when analyzing only ECGs without any of the original rhythm/conduction diagnoses for which the NN was trained (n=38 759; Figure S5).

ECG parameters were also significantly correlated with phenogroup. Increased QRS duration, PR interval, and QT interval positively correlated with phenogroup B (Figure S6).

### Genome-Wide Association Study

**Figure 5.**
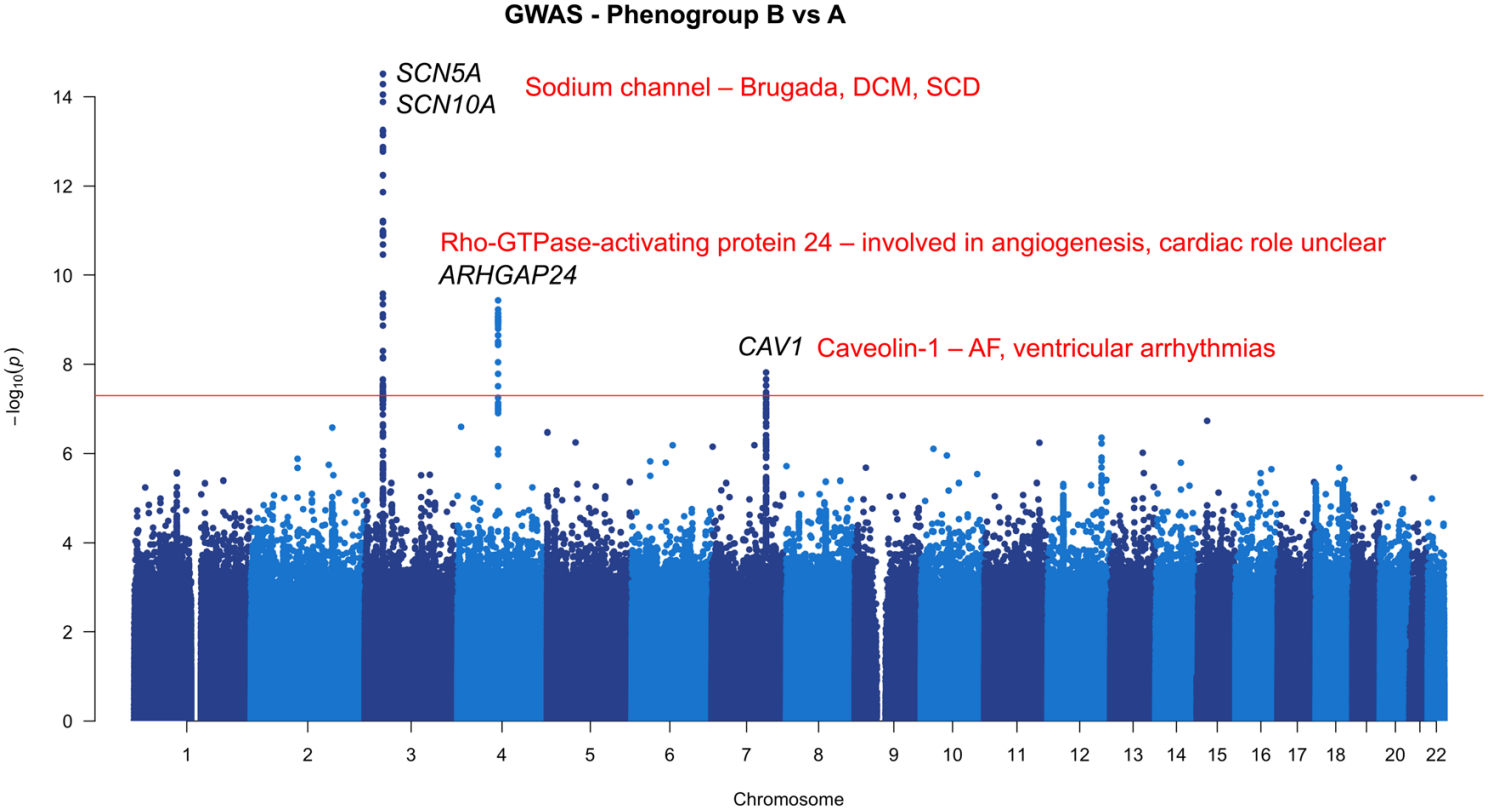
**Genome-wide association study.** Manhattan plots of genomic loci associated with ECG phenogroup. The nearest genes are annotated on the plot. The red line depicts the genome-wide significant threshold (*P*<5×10^−8^). AF indicates atrial fibrillation; DCM, dilated cardiomyopathy; and SCD, sudden cardiac death.

To identify genotypic associations of the phenogroups, a single-trait GWAS was conducted using 12.7 million variants in 31 119 subjects from the UKB. Phenogroups A and B were compared in a binary analysis. The GWAS yielded 4 loci (Figure 5), which were confirmed through expression quantitative trait loci for *SCN10A*, *SCN5A*, *CAV1*, and Rho-GTPase Activating Protein 24 (*ARHGAP24*). The lead variant for *SCN10A* has been previously significantly associated with atrial fibrillation and flutter and cardiac conduction.^22^ *CAV1* has been previously associated with QRS duration, PR interval, and QT interval, while *SCN5A* and *SCN10A* have previously well-described roles in cardiac rhythm and conduction phenotypes.^22–25^ *ARHGAP24* has been previously associated with ECG parameters including PR interval, QRS duration, and QT interval^26,27^; however, our analysis has identified for the first time *ARHGAP24* as a gene associated with a prognostically significant phenogroup. The findings were similar when analyzing only ECGs without any of the original rhythm/conduction diagnoses for which the NN was trained (Figure S7).

### Model Explainability

**Figure 6.**
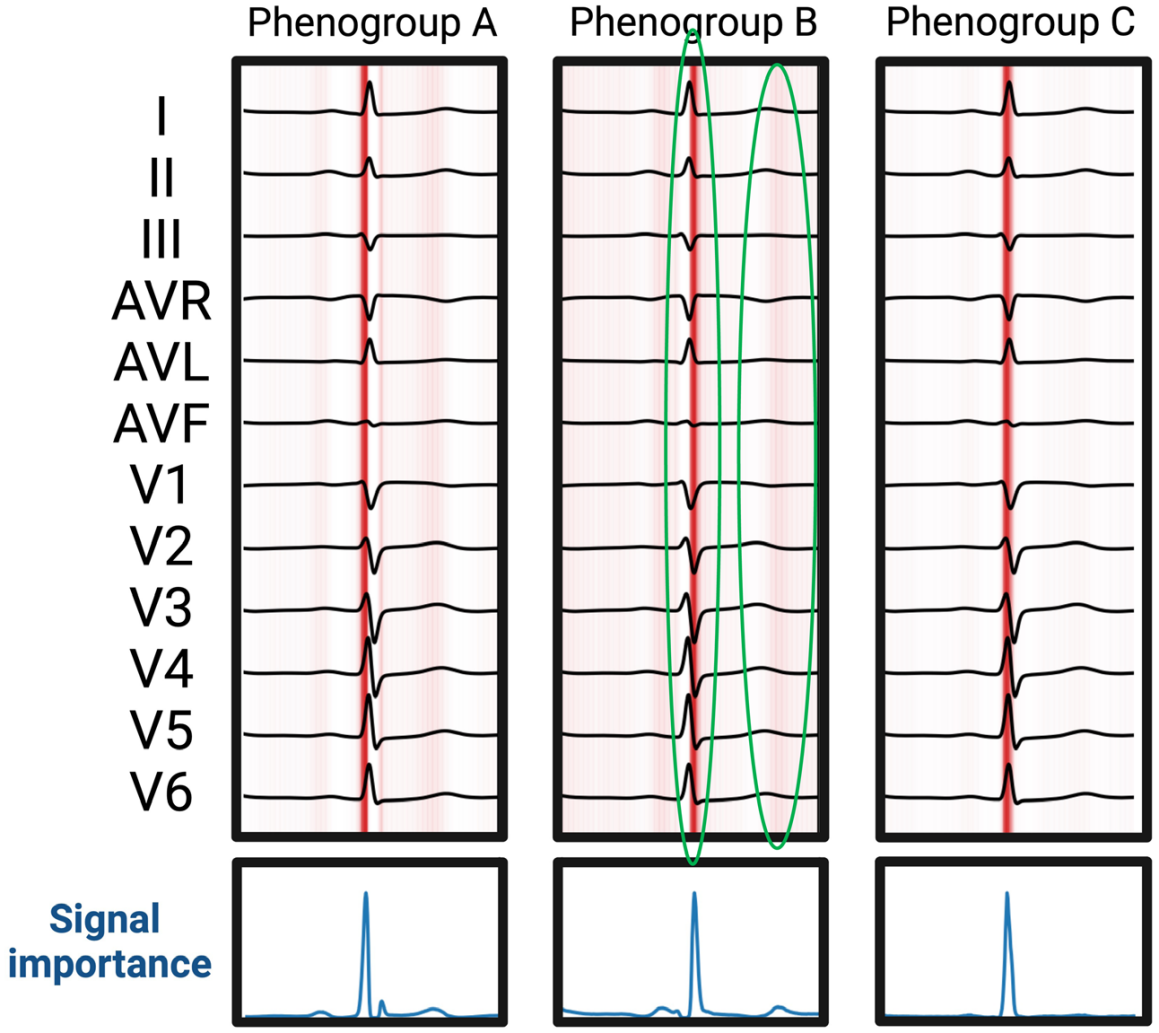
**Model explainability.** Gradient-weighted class activation mapping is used to generate importance maps showing the sections of the ECG signal deemed most important for phenogroup determination. The average saliency of 1000 ECGs from the center of each cluster is shown. Areas marked with green show the terminal QRS and terminal T wave are important for identification of the high-risk phenogroup (phenogroup B).

To better understand the reasons for phenogroup classification by the hybrid ML model, we used a modified gradient-weighted class activation mapping approach.^20^ Figure 6 shows the average saliency map for 1000 ECGs from the center of the 3 phenogroup cluster centroids. The terminal part of the QRS complex and T wave were most important for identification of the high-risk phenogroup (phenogroup B). This may reflect the importance of delayed ventricular depolarization and increased repolarization heterogeneity in explaining the increased mortality in the high-risk phenogroup.

## Discussion

In this study, we explored NN-derived ECG features to identify ECG phenogroups with prognostic significance and explored their phenotypic and genetic associations. We undertook extensive external validation across a wide range of subjects from 5 data sets—healthy volunteers to patients with cardiomyopathy, normal to abnormal ECGs, North and South American to European—and showed that the prognostic significance of the phenogroups was consistent across all data sets. Our analysis covers, to our knowledge, the largest ever mortality-linked analysis of a total of 1 810 215 subjects with robust and diverse external validation. Using PheWAS, we provide insight into the cardiac and noncardiac phenotypes associated with the phenogroups. GWAS identified genetic associations primarily related to cardiac conduction and arrhythmia but also identified a potentially novel association with ARHGAP24, which may be a pathway warranting further investigation.

### Prognostic Significance

We demonstrated that NN-derived ECG features can be used to derive phenogroups with clear prognostic significance. Although our model may, in part, be using existing ECG markers such as QRS duration, QRS microfragmentation, and bundle branch block morphology^1,2,18^ in adjusted analyses, we have shown the ECG phenogroups provide additive prognostic information beyond these.

When applied to the 3 volunteer cohorts (UKB, Whitehall II, and ELSA-Brasil) and secondary care cohort (BIDMC), phenogroup B retained its prognostic significance despite the fact that these cohorts were demographically markedly different from the derivation cohort. In contrast to these volunteer groups, the SaMi-Trop cohort provides an insight into the prognostic significance of the ECG phenogroups in a different context, in established disease in the form of Chagas cardiomyopathy, where the 3 phenogroups show marked separation in mortality curves. This analysis shows the potential for these NN-derived features and phenogroups to be applied to a diverse range of disease-specific cohorts and clinical problems, such as a broader cardiomyopathy cohort or inherited cardiac conditions such as hypertrophic cardiomyopathy.

Phenogroup C was not well represented in some populations and was almost absent from the volunteer populations. In our study, we performed external validation in diverse populations, perhaps the most diverse of any previously AI-ECG model, intentionally without any recalibration; it is, therefore, not surprising there would be some variation in model performance. Most importantly, phenogroup B retains the association with increased mortality in all external data sets.

### Biological Insights

In order for clinicians to gain actionable insights from artificial intelligence models, biological insights into model predictions are important. First, using a disease PheWAS in the BIDMC data set, we highlighted several important incident diseases that may mediate the increased mortality seen in phenogroup B, including arrhythmia, conduction disease, heart failure, and ischemic heart disease. Targeting screening and treatment toward these diseases is, therefore, 1 potential strategy for reducing risk in phenogroup B.

Second, by leveraging the extremely deep phenotyping performed in the UKB, we explored the potential biological mechanisms underlying the associations of the ECG phenogroups with survival. Our Biobank PheWAS analysis has identified reduced right and left ventricular ejection fraction and abnormalities in cardiac conduction/repolarization as potential biological mechanisms underlying the differences in prognosis of the ECG phenogroups. We report several cardiac magnetic resonance imaging associations with the higher risk phenogroup, including lower left ventricular ejection fraction, right ventricular ejection fraction, and left ventricular strain that have been previously associated with higher mortality^28–30^ and support the association with increased heart failure and cardiomyopathy seen in the disease PheWAS. Similarly, increased carotid intima-media thickness reflects atherosclerotic burden, which supports the association with increased atherosclerosis and ischemic heart disease seen in the disease PheWAS.^31^ In contrast, higher heart rate was negatively correlated with the higher risk phenogroup; this may be due to the U-shaped relationship where both low and high heart rates are associated with increased mortality.^32^

The higher-risk phenogroup was associated with increased PR interval, QRS duration, and QT interval. Increased QRS duration and QT interval have been previously associated with higher morality^33,34^; however, increased PR interval was not previously found to be associated with increased mortality.^35^ Importantly, our analyses in the UKB and Whitehall II cohorts have shown that ECG phenogroups add prognostic significance beyond basic ECG parameters.

A detailed analysis of how NN-derived features were developed throughout training was beyond the scope of this article; however, explainability analysis using gradient-weighted class activation mapping highlighted important ECG components. In particular, the terminal QRS complex, which may represent conduction slowing, and the terminal T wave, which may represent repolarization heterogeneity, were important factors in identifying the high-risk phenogroup. These factors have been previously related to adverse prognosis, which provides further reassurance to the biological relevance of our findings.^36,37^

### Genome-Wide Association Study

Phenogroups identified from NN-derived ECG features had significant genetic correlates that provide a plausible mechanistic pathway for the difference in prognosis between phenogroups. The lead variants identified here are in genes with established roles in cardiac electrophysiology: *SCN5A*, *SCN10A*, and *CAV1*.^22–25^ *SCN5A* and *SCN10A* encode the sodium channels Nav1.5 and Nav1.8, respectively. Nav1.5 is responsible for the initiation of the cardiac action potential via the inward sodium current. Mutations in *SCN5A* and *SCN10A* have been identified as a cause of inherited syndromes such as Brugada syndrome^38,39^ and dilated cardiomyopathy.^40^ A role in heart failure and sudden death in ischemic heart disease has also been described.^40,41^ *CAV1* encodes caveolin-1, which is a cytoplasmic membrane–anchored scaffolding protein that colocalizes to the atria and has been linked to atrial fibrillation, ECG parameters, and ventricular arrhythmias.^25,42,43^ *ARHGAP24* encodes Rho-GTPase–activating protein 24, which is a key regulator of angiogenesis and is involved in cell polarity, cell morphology, and cytoskeletal organization but does not have an established role in the heart.^44^ The association between *ARHGAP24* and ECG parameters has been previously described; however, the mechanism is unclear.^26,27,45^ Additionally, there is some suggestion that ARHGAP24 may be involved in human heart failure.^46^ This fits in line with our PheWAS results that implicate both the diseases of cardiomyopathy/heart failure and structural features (dilated left ventricular volumes and reduced cardiac output) with the high-risk phenogroup. The mechanisms underpinning the effect of *ARHGAP24* on the heart, therefore, warrant further exploration and may reveal novel therapeutic targets.

### Potential Clinical Applications of NN-Derived ECG Features

Using a hybrid ML approach, we have described for the first time a novel method to identify high-risk subjects from the ECG alone. Through robust and diverse external validation, we have demonstrated the relevance of the ECG phenogroups in a wide range of clinical contexts. The addition of ECG phenogroup may significantly improve the performance of risk prediction algorithms and, therefore, aid management decisions. In particular, in the higher-risk cohorts, phenogroup B had a higher risk of all-cause mortality even after adjustment for traditional cardiovascular risk factors and ECG parameters, thereby showing the additive value of this approach compared with existing strategies.

NN-derived ECG features could potentially be applied to risk stratification of a general population to identify high-risk individuals for more intensive investigation and follow-up and lower-risk individuals who may be reassured. In disease-specific cohorts such as heart failure and cardiomyopathy, NN-derived ECG features may be applied to assess risk and guide more intensive medical therapy, implantable devices, or transplantation consideration. These potential applications, however, require further evaluation in the appropriate populations in prospective studies.

Age and sex attenuate the performance of the ECG phenogroup as a singular risk biomarker. This is likely, in part, due to the ability of the ECG to capture biological age (10 years). Age, in particular, is likely to be one of the biggest predictors of mortality in any individual, so this is, therefore, not surprising. Previous work has shown age and sex alone can be used to predict 5-year mortality with impressive performance (accuracy, 71.6% and area under the receiver operating characteristic curve, 0.78).^47^ Any clinical application of our model would, therefore, include age as a covariate in a multivariate risk prediction model.

### Limitations

We have described the novel application of NN-derived ECG features for risk prediction and externally validated our findings in a diverse population. The volunteer cohorts of the UKB, Whitehall II, and ELSA-Brasil are selected populations. This may explain the absence of phenogroup C in these cohorts. It is possible that the absence of phenogroup C in these external data sets represents reduced applicability of this model in other data sets. This would not be surprising given that the tested data sets are extremely diverse. The Biobank PheWAS and GWAS analyses were performed in a population with predominantly European ancestry (UKB), and findings, therefore, may not be generalizable globally. Although the phenogroups described in this article are associated with adverse prognosis and important cardiovascular diseases, we have not compared this approach directly with a supervised ML approach, which may more accurately identify individuals at high risk of death. Due to the large data set size, large number of features per ECG, and detailed downstream phenogroup exploration performed, a detailed exploration of other unsupervised ML methodologies was out of the scope of this article. Other unsupervised ML methods may identify different phenogroups.

### Conclusions

We describe the use of NN-derived ECG features, derived from an established AI-ECG model trained to identify 6 common diagnoses, to identify prognostically significant phenogroups from the 12-lead ECG. We explored the biological basis underlying the difference in prognosis between the phenogroups and identified phenotypic and genotypic associations through PheWAS and GWAS. We validated our findings in 5 external data sets across 2 continents and diverse patient populations. NN-derived ECG features have important applications beyond the original model from which they are derived and may be transferable and applicable for risk prediction in a wide range of settings, in addition to mortality prediction.

## Article Information

### Acknowledgments

This study has been conducted using the UK Biobank Resource under application numbers 48666 and 47602. The authors also thank InSIGHT Core in the Center for Healthcare Delivery Science at Beth Israel Deaconess Medical Center for assistance in obtaining primary data.

### Sources of Funding

Dr Sau is funded by a British Heart Foundation (BHF) clinical research training fellowship (FS/CRTF/21/24183). Drs Ng and Peters are supported by the BHF (RG/F/22/110078). Dr Ng is also supported by the National Institute for Health Research Imperial Biomedical Research Centre. Dr O’Regan is supported by the Medical Research Council (MC_UP_1605/13) and National Institute for Health Research Imperial College Biomedical Research Centre and the British Heart Foundation (RG/19/6/34387 and RE/18/4/34215). Drs Andršová, Novotný, and, partially, Malik have been supported by Ministry of Health of the Czech Republic (FNBr, 65269705). AS, LP, FSN, AR and ALPR are supported by the Academy of Medical Sciences NGR1\1746. The authors acknowledge support from Imperial’s BHF Centre for Excellence Award (RE/18/4/34215 and RE/24/130023).

### Disclosures

None.

### Supplemental Material

Supplemental Methods

Tables S1–S4

Figures S1–S7

Supplemental Results

References 48–56

## Supplementary Material


