# Supplementary material for: Prognostic Significance and Associations of Neural Network–Derived Electrocardiographic Features
Source: Circ Cardiovasc Qual Outcomes. 2024 Nov 14;17(12):e010602. doi: 10.1161/CIRCOUTCOMES.123.010602 (PMC7616866; doi:10.1161/CIRCOUTCOMES.123.010602)

Neural network-derived electrocardiographic features have prognostic significance and important phenotypic and genotypic correlates

SUPPLEMENTAL MATERIAL

Supplementary methods

Ethical approvals

The Clinical Outcomes in Digital Electrocardiography (CODE) study was approved by the Research Ethics Committee of the Universidade Federal de Minas Gerais, protocol 49368496317.7.0000.5149. The Whitehall II study was approved by the Joint University College London/University College London Hospitals Committees on the Ethics of Human Research. The UK Biobank has approval from the North West Multi-Centre Research Ethics Committee as a Research Tissue Bank (application ID 48666). The Longitudinal Study of Adult Health (ELSA-Brasil) was approved by the Research Ethics Committees of the participating institutions and by the National Committee for Research Ethics (CONEP 976/2006) of the Ministry of Health. The São Paulo-Minas Gerais Tropical Medicine Research Center (SaMi-Trop) study was approved by the Brazilian National Institutional Review Board (CONEP), No. 179.685/2012. For the Beth Israel Deaconess Medical Center (BIDMC) cohort ethics review and approval was provided by the Beth Israel Deaconess Medical Center Committee on Clinical Investigations, IRB protocol # 2023P000042.

ECG datasets

(i) The CODE Cohort

The CODE cohort is a database of 2,322,513 ECG records from 1,676,384 different patients of 811 counties in the state of Minas Gerais/Brazil from the Telehealth Network of Minas Gerais (TNMG). The cohort is linked to public mortality databases. Patients over 16 years old with a valid ECG performed from 2010 to 2017 were included. Clinical data were self-reported. In a 15% stratified sample of the original cohort (CODE-15) ECGs were labelled by diagnosis. We analysed the “normal” ECGs according to conventional clinical reporting and based on automated interval measurements [10].

(ii) ELSA-Brasil Cohort

ELSA-Brasil is a cohort study of 15,105 Brazilian public servants, aged 35 to 74 at enrolment. All active or retired employees of six participating institutions were eligible for the study. The full inclusion criteria and protocol have been previously described [13]. 13,739 subjects had ECG and outcome data available for analysis.

(iii) The SaMi-Trop Cohort

The SaMi-Trop cohort is a prospective cohort of 1,631 patients with chronic Chagas cardiomyopathy and has been previously described in detail [14]. Briefly, the inclusion criteria were: (1) self-reported Chagas disease; (2) aged 19 years or more. Digital ECGs were performed in 2011-2012 by TNMG. 83% of this cohort had abnormal ECGs [21].

(iv) The UK Biobank Cohort

The UK Biobank is longitudinal study of over 500,000 volunteers aged 40-69 at the time of enrolment in 2006-2010 [12]. At baseline assessment participants provided information on health and lifestyle via questionnaire, had physical measures taken (including height, weight, and blood pressure) and donated samples of blood urine and saliva. A subgroup of participants were invited back for subsequent visits for additional investigations, including for detailed studies including cardiac magnetic

resonance imaging (MRI), brain MRI and digital ECGs. 42,386 subjects with digital ECGs taken at the instance 2 visit were available for analysis. There is evidence of healthy volunteer selection bias [48]. Outcomes were linked to cancer and death registry data, hospital admissions and primary care records. Major adverse cardiovascular events (MACE) was selected as the primary endpoint for this dataset given the low mortality rate in this healthy population and the additional data available. MACE was defined as: heart attack, stroke, heart failure diagnosis and all cause death. Detailed phenotyping using the cardiac MRI data has been previously described [19,49]. Cause of death was ascertained based on the ICD10 code stated as the primary cause of death

(v) The Whitehall II Cohort

The Whitehall II cohort has been previously described [11]. Briefly, British civil servants were enrolled into this cohort and had repeated calls for medical investigations. Between 2007 and 2009 participants (n=5,066) had digital 12-lead ECGs performed, for 5 minutes. The first 10s of this recording was considered in our analysis. Causes of death were ascertained based on the information contained in the death certificate. The novel ECG parameters micro-QRS fragmentation and QRS-T angle have been previous applied to this cohort, the same values were used in our analysis [18].

(vi) The BIDMC cohort

The BIDMC cohort is a dataset comprised of routinely collected data from Beth Israel Deaconess Medical Center, Boston, USA. Subject over 16 years old with a valid ECG performed from 2000 to 2023 were included. ECGs were linked to mortality records. The first ECG recorded per subject was used in this analysis. 188,972 subjects were available for analysis. Cardiovascular death in the BIDMC cohort was defined as a mortality occurring within 30 days of a diagnostic code for acute myocardial infarction, ischaemic stroke, intracranial haemorrhage, sudden cardiac death, or heart failure as previously described [50,51].

Unsupervised machine learning for clustering into phenogroups

The sklearn package in Python was used to perform K-means clustering [52]. In order to determine cluster assignment in the external validation cohorts, the 5120 features were extracted using CODE-CNN as described in the main manuscript methods. The K-means model (trained on the NN-derived ECG features from the CODE dataset) was then used to identify the nearest cluster centre and therefore determine the cluster assignment for each ECG. There was no retraining or calibration performed when applying the CODE-CNN and K-means models to the external validation datasets.

Survival analysis in derivation and external validation cohorts

Survival analysis was repeated after excluding subjects with the six diagnoses identified by the CODE-CNN and also considering only physician-adjudicated normal ECGs in the CODE dataset. Descriptive statistics are displayed as medians (interquartile ranges) for continuous variables and numbers (percentages) for categorical variables. Kaplan-Meier plots were used to display cumulative mortality. The log rank test was used to compare survival curves.

Cox proportional hazards regression modelling was used to estimate hazard ratios for mortality while correcting for other known variables. Recent work suggests virtually all real-world clinical datasets will violate the proportional hazards assumptions if sufficiently powered and that statistical tests for the proportional hazards assumption may be unnecessary [53]. In line with these recommendations, the proportional hazards assumption was not evaluated and the hazard ratio from our Cox models should be interpreted as a weighted average of the true hazard ratios over the follow-up period. The adjusted variables differed in each dataset depending on the data available. Statistical analyses were performed with R 4.0.0 statistical package (R Core Team, Vienna, Austria) or Python (version 3.9).

Phenome-wide association study

We used two PheWAS approaches, firstly we used a Disease PheWAS to explore the association of ECG phenogroup with incident diseases and treatments. Using the BIDMC dataset we converted International Classification of Diseases (ICD) 9 and 10 codes into Phecodes as previously described [54]. In order to remove prevalent disease, we removed any incident codes if ICD codes prior to the ECG also existed. Phecodes with under 20 cases were excluded. Logistic regression was performed to investigate the association between ECG phenogroup and incident disease. The second approach, Biobank PheWAS, used the UK Biobank that contains data from over 3000 phenotypes derived from patient measurements, surveys, and investigations. Univariate correlation was performed to investigate the association between ECG phenogroup and phenotypes. Highly correlated features (>95%), features with >90% missing data or with >95% of exactly the same value were discarded. We adjusted for multiple testing using Bonferroni correction. Given the very small number of subjects in phenogroup C, subjects in phenogroup C were excluded from these analyses. Effects of age and sex were regressed out of the ECG phenogroup variable. PheWAS analyses were performed in Python (version 3.9).

Genome-wide association study

Standard quality control was undertaken. Included single nucleotide polymorphisms (SNPs) had a minor allele frequency (MAF) >0.1% and an imputation INFO score of >0.4. Unrelated individuals of genetically-determined European ancestry were included as previously described [55]. The GWAS was undertaken using FastGWA generalized linear mixed model association analysis through the Genome-wide Complex Trait Analysis software using a genetic relationship matrix (GRM) to adjust for population structure [56]. The Manhattan plots depict the nearest gene. Top SNPs were identified that had a P-value <5x10-8. Associations of the detected SNPs were evaluated using the NHGRI-EBI GWAS Catalog, PhenoScanner, GTEx, and GeneAtlas UK Biobank PheWAS browser.

Model explainability

A signal importance map for a given input of a 12-lead ECG signal was computed. First, the final layer of the original trained CNN is removed, and a new layer with a fixed operation is added. This layer takes the 5120 features from the trained CNN

and computes its Euclidean distance to the centre of three phenogroup clusters, which results in three distance scores. Using softmin, these distance scores are mapped to probability scores. Using Grad-CAM, for a given input, we compute the gradient of weights of the first convolution layer with respect to the probability score for a specified phenogroup (e.g., phenogroup A). The output of the first layer for a given input is then multiplied by an average gradient of respective channels, and finally, the positive values are taken as the importance score for the saliency map. To extract the generalised saliency map for each phenogroup, 1000 ECGs from the centre of the three phenogroup clusters (approximately equidistant from all three cluster centroids) were taken. After computing saliency maps, each 10s ECG was averaged to one beat per lead by aligning beats with R-peaks. Similarly, the respective saliency map of each 10s was averaged to a single beat. A generalised saliency map for a cluster was computed by averaging (median) all the 1000 saliency maps. While plotting the generalised saliency map, single beats of all the 1000 ECGs were also averaged (mean) to one beat per lead.

Supplementary Results

Further analysis of Phenogroup C

First, using logistic regression we investigated the significant clinical predictors of assignment to phenogroup C vs A. The presence of hypertension (OR 6.92, p < 0.0001), younger age (OR 0.98 per year, p < 0.0001), and tobacco use (OR 3.78, p < 0.0001) were the strongest clinical differentiators. Data on CV risk factors was not available in Sami-TROP, however younger age was a significant predictor of Phenogroup C assignment (OR 0.97 per year, p < 0.0001). Phenogroup C therefore represents a younger cohort with increased cardiovascular risk (CV) factors. It is possible that the younger age counteracts the increased CV risk factors, leading to a mortality profile similar to Phenogroup A.

We then investigated the boundaries of the phenogroups in the volunteer cohorts of the UK Biobank and ELSA-Brasil, where data on cardiovascular risk factors was available. Specifically, using the distance to Phenogroup C, we reassigned the third of the each cohort that was closest to the Phenogroup C centroid. We found that the reassigned Phenogroup C had a similar mortality profile to Phenogroup A (ELSA Phenogroup C vs A: Unadjusted HR 1.20 (0.997-1.45), p 0.053. UK Biobank Phenogroup C vs A: Unadjusted HR 1.17 (1.00-1.38, p = 0.048)), this was consistent with the mortality profile of Phenogroup C in CODE, which was similar to Phenogroup A.

Next, using logistic regression we investigated the significant clinical predictors of assignment to reassigned Phenogroup C vs A. In these analyses, age had the opposite direction of effect, with older subjects more likely to be reassigned to Phenogroup C ((ELSA OR 1.02, p < 0.0001. UKB OR 1.01 per year, p < 0.0001). Hypertension (ELSA OR 1.20, p = 0.37. UKB OR 1.00, p = 0.99) and smoking status (ELSA OR 1.05, p 0.39. UKB OR 1.09, p = 0.24) were not significant predictors.

We therefore conclude that Phenogroup C was a population of younger subjects with increased cardiovascular risk factors that was not well represented in all populations. Reassignment of subjects nearest the Phenogroup C centroid showed similar survival properties to Phenogroup A, however the determinants of Phenogroup C assignment in CODE did not hold true in the reassigned Phenogroup C subjects (from Phenogroup A); suggesting these subjects should not be assigned to Phenogroup C, and their original assignments were more suitable.

Further analysis of location within centroid

We additionally examined position within the high-risk centroid to evaluate if this had an additional prognostic value beyond phenogroup assignment alone. We further divided phenogroup B into three groups based on proximity to the phenogroup B centroid. We found the group furthest from the phenogroup B centroid had the highest mortality risk when compared to phenogroup A (Furthest group unadjusted HR 3.28 (3.17-3.39), middle group HR 2.70 (2.62- 2.78), closest group 2.36 HR (2.30-2.42)). Furthermore we divided phenogroup B by proximity to the centre of all three centroids, and found the subgroup furthest from the centre of the centroids had the highest mortality risk (Furthest group HR 3.27 (3.19-3.35), middle group HR 1.78 (1.73-1.84), closest group unadjusted HR 1.96 (1.89-2.04)). Overall these findings

suggest the ECGs on the outer periphery of overall distribution but closest to phenogroup B, had the highest risk profile.

Tables
Table S1
Pairwise distances between cluster centroids

	A	B	C
A	-	0.248	0.257
B	0.248	-	0.314
C	0.257	0.313	-

Table S2
Dataset demographics

	Phenogroup A	Phenogroup B	Phenogroup C	P value
**CODE dataset**				
n	450,336	481,026	627,059	
Age (mean (SD))	48.68 (16.81)	58.20 (17.69)	48.78 (16.68)	<0.001
Sex (M, n (%))	167,730 (37.2)	238,029 (49.5)	221,283 (35.3)	<0.001
Hypertension (n (%))	43,060 (9.6)	185,738 (38.6)	263,842 (42.1)	<0.001
Previous MI (n (%))	999 (0.2)	5,543 (1.2)	5,062 (0.8)	<0.001
Smoker (n (%))	10,290 (2.3)	34,927 (7.3)	63,598 (10.1)	<0.001
Diabetes mellitus (n (%))	9,318 (2.1)	35,684 (7.4)	56,468 (9.0)	<0.001
COPD (n (%))	1,137 (0.3)	4,280 (0.9)	5,849 (0.9)	<0.001
Chagas disease (n (%))	1,234 (0.3)	21,187 (4.4)	12,169 (1.9)	<0.001
Hyperlipidaemia (n (%))	5,007 (1.1)	22,332 (4.6)	33,251 (5.3)	<0.001
1st degree AVB (n (%))	237 (0.1)	20,470 (4.3)	141 (0.0)	<0.001
RBBB (n (%))	100 (0.0)	37,292 (7.8)	22 (0.0)	<0.001
LBBB (n (%))	147 (0.0)	20,447 (4.3)	16 (0.0)	<0.001
Sinus tachycardia (n (%))	52 (0.0)	34,201 (7.1)	116 (0.0)	<0.001
Sinus bradycardia (n (%))	58 (0.0)	24,467 (5.1)	40 (0.0)	<0.001
Atrial fibrillation (n (%))	8 (0.0)	20,671 (4.3)	43 (0.0)	<0.001
**Whitehall II**				
n	3,370	1,696	*	<0.001
Age (mean (SD))	64.99 (5.63)	66.48 (5.89)		<0.001
Sex (M, n (%))	2,236 (66.4)	1,365 (80.4)		<0.001
Heart rate (bpm, mean (SD))	70.48 (9.47)	62 (13.54)		<0.001
QTc interval (ms, mean (SD))	423.53 (17.59)	425.59 (29.59)		<0.001
QRS duration (ms, mean (SD))	104.85 (8.4)	111.12 (18.28)		<0.001
**UK Biobank**				
n	25,723	16,466	197	
Age (mean (SD))	63.47 (7.60)	65.22 (7.85)	60.37 (7.59)	<0.001
Sex (M, n (%))	10,558 (41.0)	9,957 (60.5)	23 (11.7)	<0.001
Heart rate (bpm, mean (SD))	65.67 (8.72)	56.00 (11.22)	74.21 (5.80)	<0.001
QRS duration (ms, mean (SD))	85.42 (9.73)	93.86 (18.10)	80.21 (8.96)	<0.001
PR interval (ms, mean (SD))	156.61 (19.34)	179.41 (32.44)	141.98 (12.96)	<0.001
QTc interval (ms, mean (SD))	423.78 (22.24)	417.31 (30.04)	433.26 (19.37)	<0.001
**ELSA Brasil**				
n	11,175	2,536	*	
Age (mean (SD))	51.69 (8.87)	54.83 (9.67)		<0.001
Sex (M, n (%))	4771 (42.7)	1477 (58.2)		<0.001
BMI (mean (SD))	27.01 (4.77)	27.07 (4.69)		0.625
Obesity (n (%))	2571 (23.0)	572 (22.6)		0.643
Previous MI (n (%))	149 (1.3)	102 (4.0)		<0.001

Heart failure (n (%))	135 (1.2)	92 (3.6)		<0.001
Previous stroke (n (%))	139 (1.2)	49 (1.9)		0.009
Hypertension (n (%))	3,799 (34.0)	1,120 (44.2)		<0.001
Diabetes mellitus (n (%))	2,218 (19.8)	505 (19.9)		0.964
Coronary artery disease (n (%))	264 (2.4)	177 (7.2)		<0.001
Hyperlipidaemia (n (%))	5,549 (49.7)	1,253 (49.4)		0.853
Smoker (n (%))	1,461 (13.1)	337 (13.3)		0.799
**Sami-TROP**				
n	286	1,130	215	
Age (mean (SD))	60.25 (12.73)	60.04 (12.86)	54.88 (11.60)	<0.001
Sex (M, n (%))	80 (28.0)	411 (36.4)	43 (20.0)	<0.001
**BIDMC**				
n	118602	70245	125	
Age (mean (SD))	52.15 (16.99)	61.51 (18.59)	40.47 (16.16)	<0.001
Sex (M, n (%))	51633 (43.5)	38832 (55.3)	40 (32.0)	<0.001

Categorical variables n (%), continuous variables mean (SD)
*Only 5 subjects were in phenogroup C in the Whitehall II cohort, and 28 in the ELSA Brasil Cohort, therefore further analysis was not performed in these groups.

MI: myocardial infarction, COPD: chronic obstructive pulmonary disease, AVB: atrioventricular block, RBBB: right bundle branch block, LBBB: left bundle branch block. CODE: Clinical Outcomes in Digital Electrocardiography, UKB: UK Biobank, BIDMC: Beth Israel Deaconess Medical Center, SaMi-Trop: São Paulo-Minas Gerais Tropical Medicine Research Center, ELSA-Brasil: Brazilian Longitudinal Study of Adult Health

Table S3
Cox regression models for prediction of mortality (unless otherwise specified)

	Adjusted		
Analysis	HR	95% CI	P value
**CODE***			
Cluster B	1.20	1.17-1.23	<0.0001
Cluster C	0.82	0.80-0.84	<0.0001
**CODE* – no ECG diagnosis cohort**			
Cluster B	1.15	1.12-1.19	<0.0001
Cluster C	0.80	0.78-0.83	<0.0001
**CODE* – normal ECG cohort**			
Cluster B	0.94	0.80-1.10	0.42
Cluster C	0.95	0.84-1.09	0.49
**UKB (MACE)** †			
Cluster B	1.21	1.01-1.45	0.04
**UKB (all-cause mortality)** †			
Cluster B	1.15	0.90-1.46	0.27
**Whitehall II (all-cause mortality)** ‡			
Cluster B	1.61	1.16-2.24	0.005
**Whitehall II (cardiovascular mortality)** ‡			
Cluster B	1.72	0.92-3.18	0.09
**Whitehall II (non-cardiovascular mortality) ‡**			
Cluster B	1.54	1.05-2.24	0.03
**ELSA-Brasil (all-cause mortality)** §			
Cluster B	1.04	0.86-1.27	0.65
**SaMi-Trop** ‖			
Cluster B	1.50	0.85-2.64	0.16
Cluster C	0.50	0.17-1.54	0.23
**BIDMC** #			
Cluster B	1.20	1.17-1.24	<0.0001
Cluster C	0.73	0.33-1.63	0.45

CODE no ECG diagnosis cohort: None of the following diagnoses on the ECG: 1st degree AVB, RBBB, LBBB, sinus tachycardia, sinus bradycardia and atrial fibrillation

* Adjusted for age, sex, hypertension, previous MI, smoking status, diabetes mellitus, COPD, chagas disease, hyperlipidaemia, 1st degree AVB, RBBB, LBBB, sinus tachycardia, sinus bradycardia and atrial fibrillation
† Adjusted for age, sex, previous MI, smoking status, hypertension, diabetes mellitus, hyperlipidaemia, PR interval, QRS duration, QT interval, QTc interval and heart rate
‡ adjusted for age, sex, heart rate, QTc interval, QRS duration, $\log_2$ (QRS micro-fragmentation) and total cosine R to T
§ adjusted for age, sex, hypertension, previous MI, smoking status, diabetes mellitus, BMI, obesity, heart failure, previous stroke, coronary artery disease and hyperlipidaemia
II adjusted for age and sex
adjusted for age, sex, diabetes mellitus, smoking status, hypertension, hyperlipidaemic, heart rate, PR interval, QRS duration, QTc interval

HR: Hazard ratio, CODE: Clinical Outcomes in Digital Electrocardiography, UKB: UK Biobank, BIDMC: Beth Israel Deaconess Medical Center, SaMi-Trop: São Paulo-Minas Gerais Tropical Medicine Research Center, ELSA-Brasil: Brazilian Longitudinal Study of Adult Health. MACE: Major adverse cardiovascular event, MI: myocardial infarction, COPD: chronic obstructive pulmonary disease, AVB: atrioventricular block, RBBB: right bundle branch block, LBBB: left bundle branch block.

Table S4

Sub-analysis by cause of death in BIDMC and UKB

Analysis	HR	95% CI	P value
**BIDMC - cardiovascular mortality**			
Cluster B	3.22	3.07-3.38	< 0.0001
Cluster C	0.39	0.055-2.80	0.351
**BIDMC - non-cardiovascular mortality**			
Cluster B	2.36	2.31-2.42	< 0.0001
Cluster C	0.39	0.16-0.95	0.0374
**UKB - cardiovascular mortality**			
Cluster B	2.63	1.78-3.88	< 0.0001
**UKB - non-cardiovascular mortality**			
Cluster B	1.21	(0.996-1.47)	0.055

UKB: UK Biobank, BIDMC: Beth Israel Deaconess Medical Center, HR: hazard ratio

Figure S1
Elbow plot for selection of k, dashed line indicates the elbow

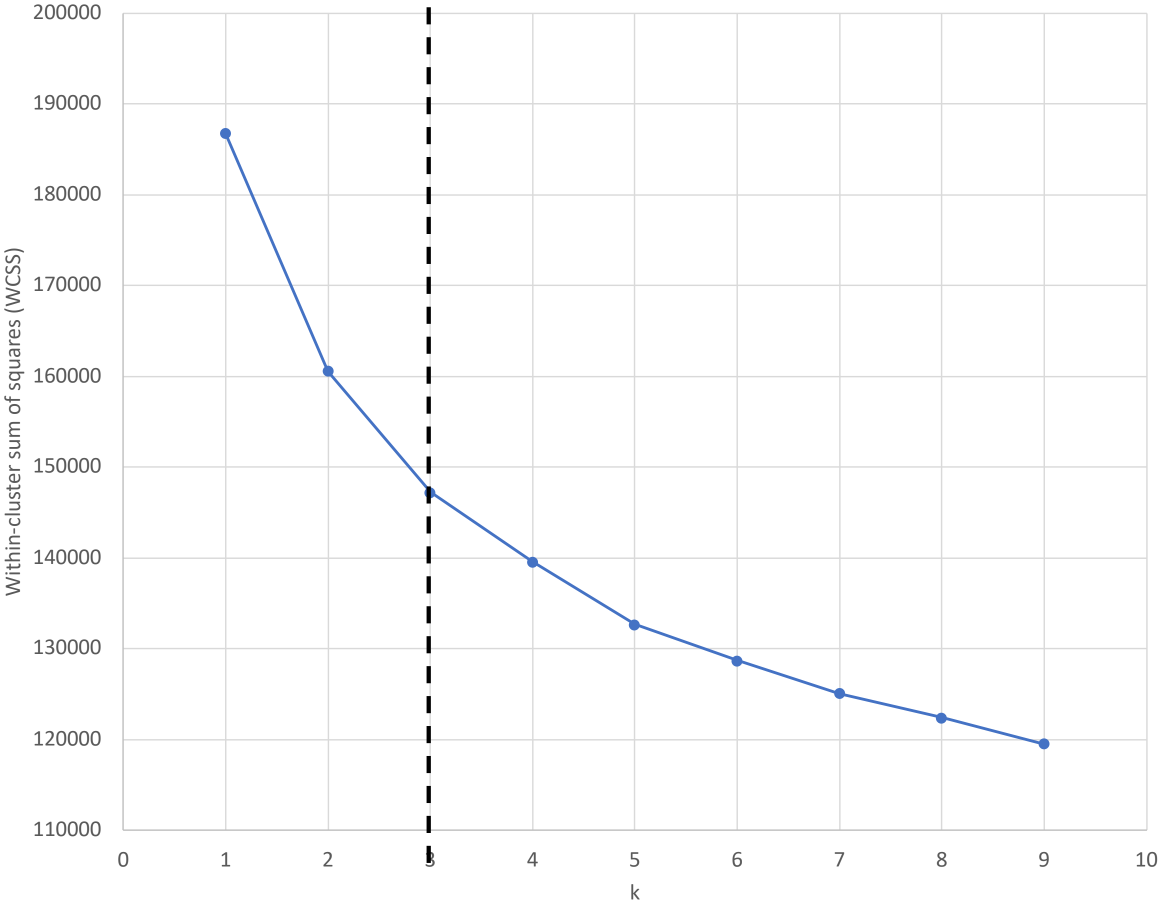

Figure S2
Cluster visualisation, PCA is applied following by TSNE using the top 50 principal components.
CODE: Clinical Outcomes in Digital Electrocardiography, UKB: UK Biobank, BIDMC: Beth Israel Deaconess Medical Center, SaMi-Trop: São Paulo-Minas Gerais Tropical Medicine Research Center, ELSA-Brasil: Brazilian Longitudinal Study of Adult Health

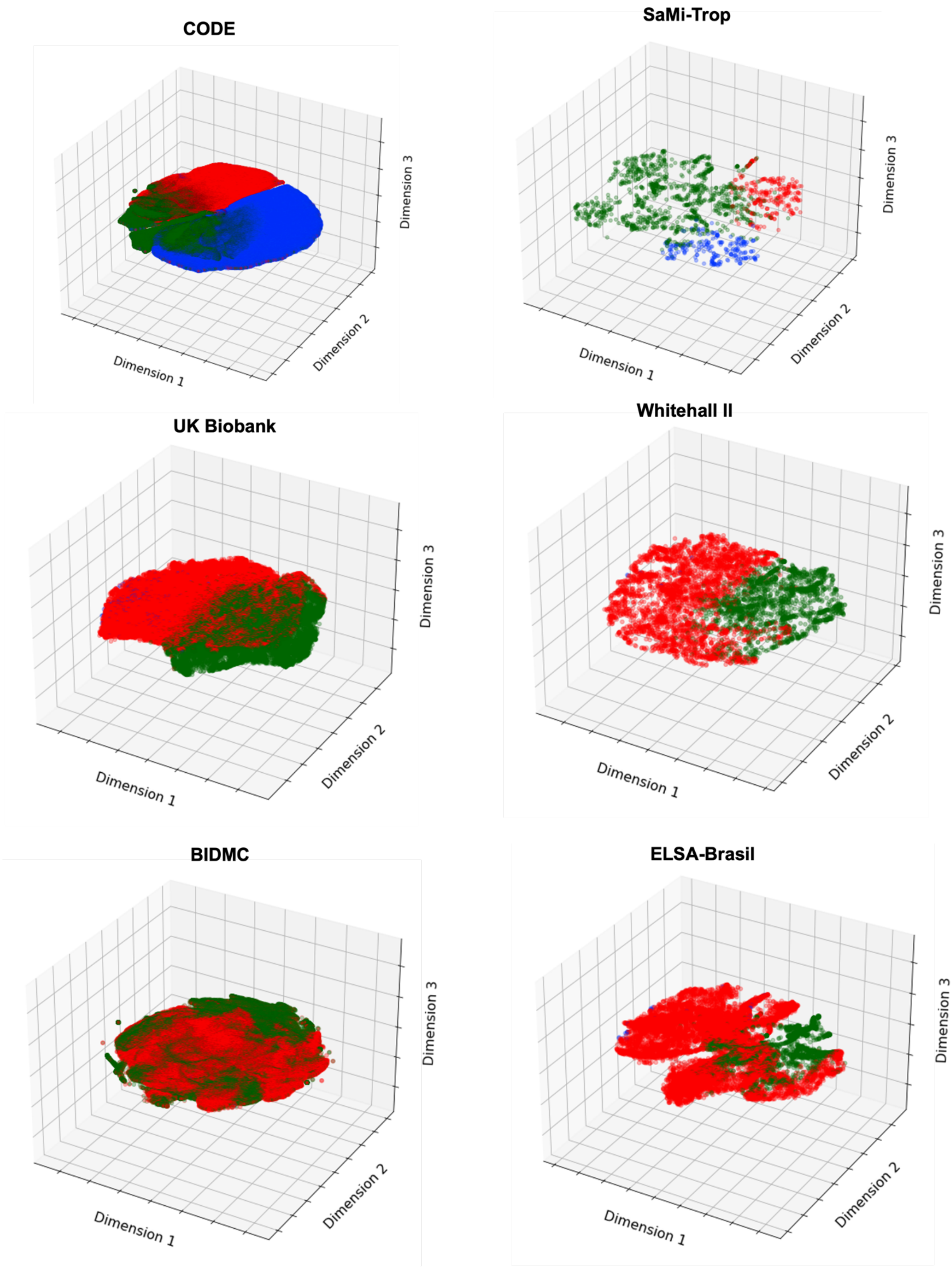

Figure S3

Sensitivity analysis with values of k from 2 to 9

K = 3 was selected based on the elbow plot shown above. Sensitivity analyses were performed to explore different values of K. This exploratory analysis supports the selection of K = 3. Higher numbers of K do not appear to highlight phenogroups with significantly differential prognosis. K = 2 resulted in a smaller risk difference between the high and low risk group compared to K = 3 (unadjusted HR: K = 2: 2.54 (2.49-2.59), K = 3: 3.03 (2.97- 3.10)).

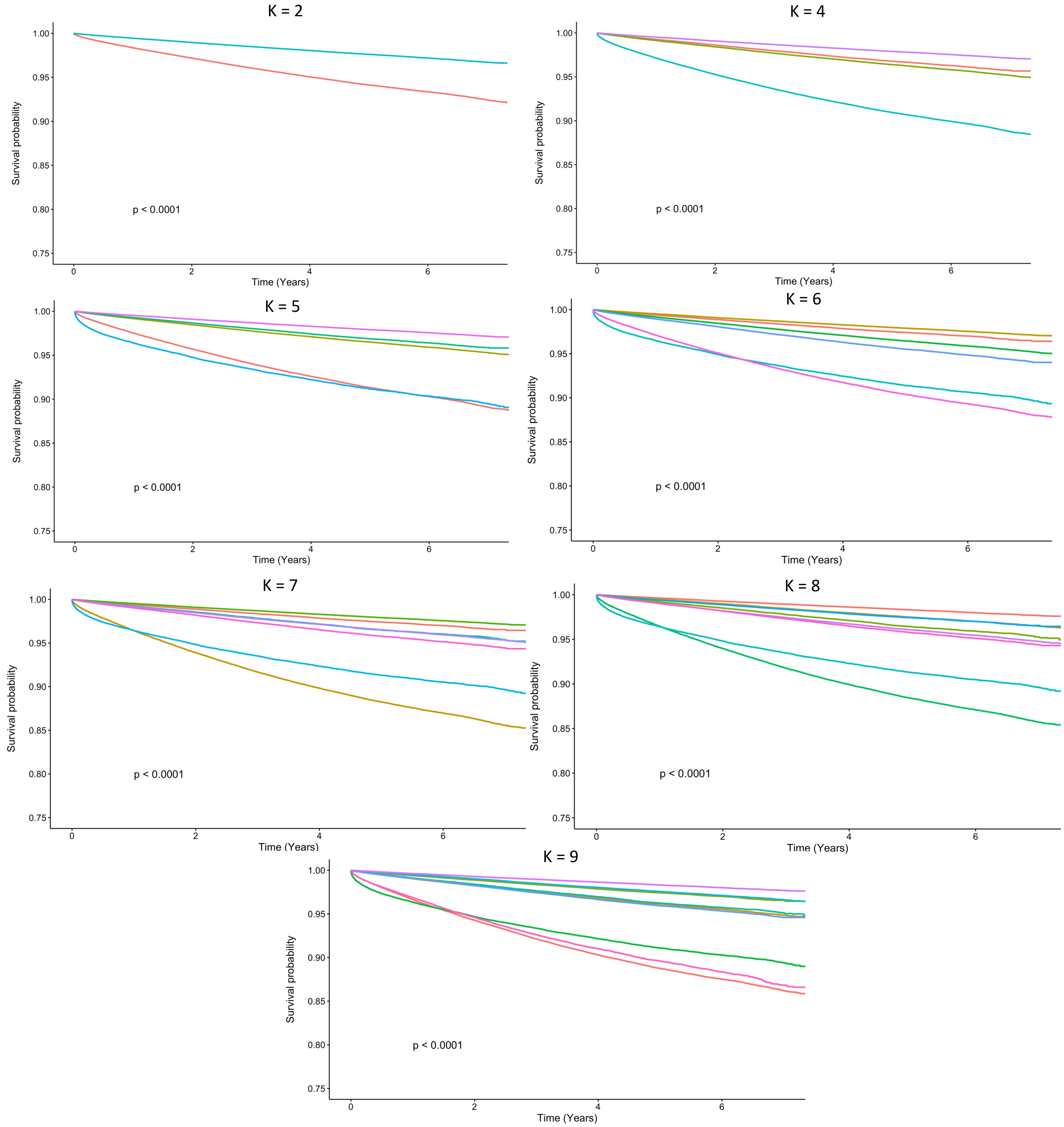

Figure S4

Survival analysis in the four external validation datasets with removal of subjects with any of the following diagnoses on the ECG: 1st degree AVB, RBBB, LBBB, sinus tachycardia, sinus bradycardia and atrial fibrillation. In the volunteer populations (Whitehall II, UK Biobank and ELSA-Brasil) phenogroup C has very few subjects and therefore is excluded. (A) Whitehall II (B) UK Biobank (survival free of major adverse cardiovascular events is depicted) (C) ELSA-Brasil cohort (D) SaMi-TROP cohort and (E) BIDMC cohort. Phenogroup B has a significantly higher event rate. CODE: Clinical Outcomes in Digital Electrocardiography, UKB: UK Biobank, BIDMC: Beth Israel Deaconess Medical Center, SaMi-Trop: São Paulo-Minas Gerais Tropical Medicine Research Center, ELSA-Brasil: Brazilian Longitudinal Study of Adult Health

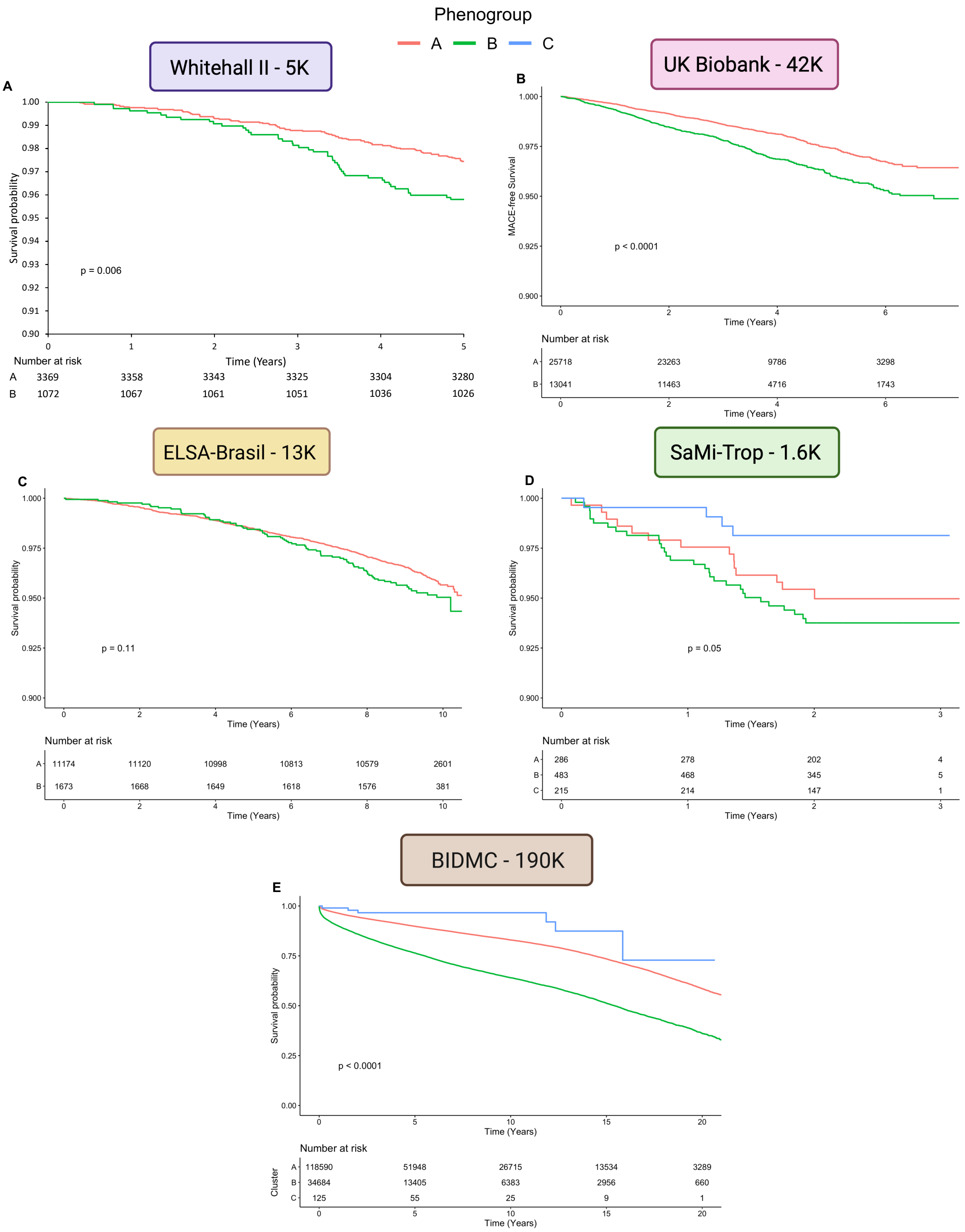

Figure S5

UK Biobank Phenome-wide association study (PheWAS) secondary analysis. Subset of patients without any of the following diagnoses on the ECG: 1st degree AVB, RBBB, LBBB, sinus tachycardia, sinus bradycardia and atrial fibrillation. AVB: atrioventricular block, RBBB: right bundle branch block, LBBB: left bundle branch block

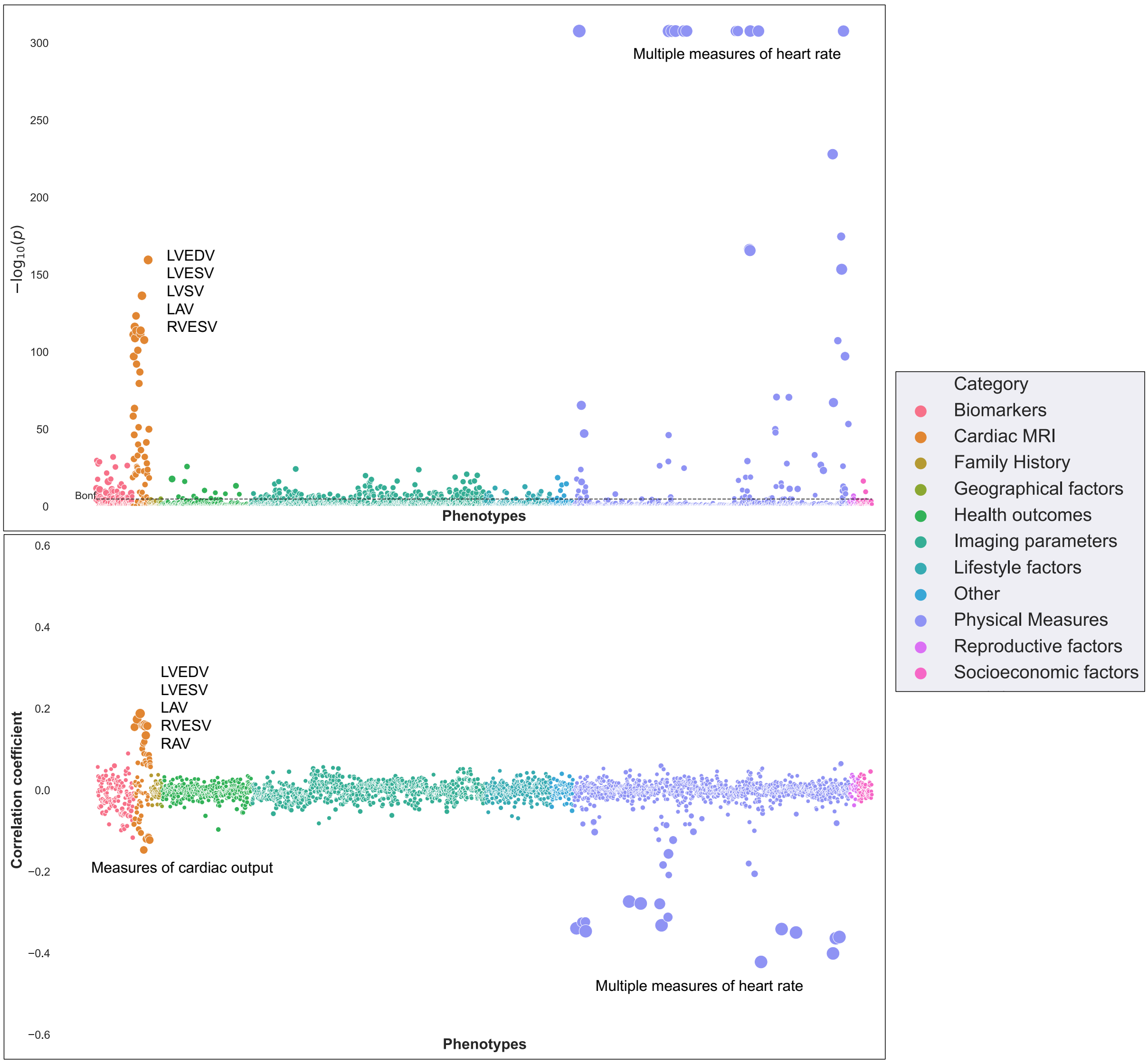

Figure S6

UK Biobank Phenome-wide association study (PheWAS) subplot depicting correlation coefficients of ECG parameter results

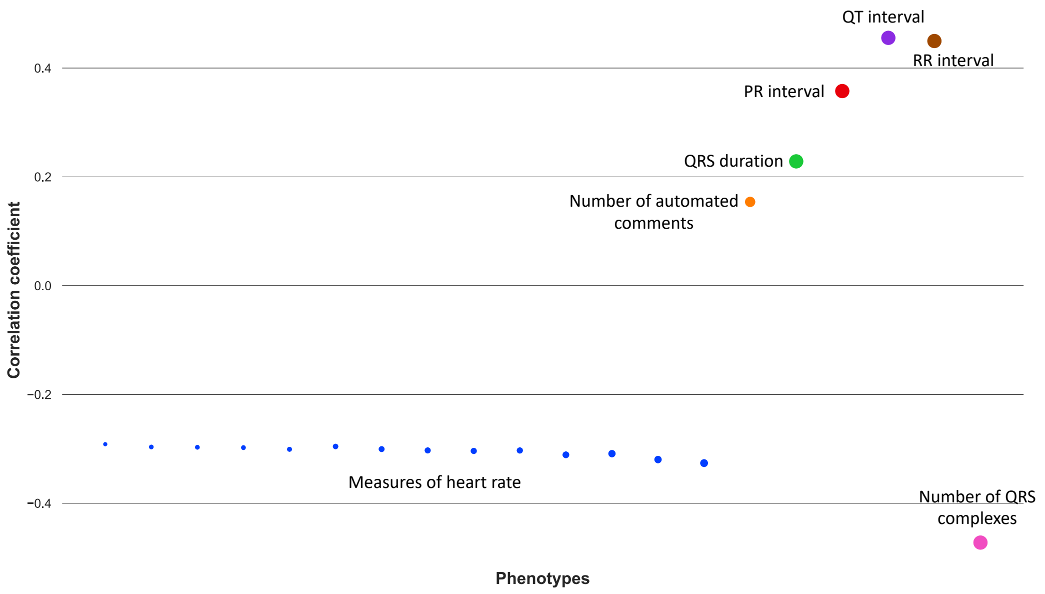

Figure S7
GWAS secondary analysis. Subset of patients without any of the following diagnoses on the ECG: 1st degree AVB, RBBB, LBBB, sinus tachycardia, sinus bradycardia and atrial fibrillation.
Manhattan plots of genomic loci associated with ECG phenogroup. SNPs with significant associations with the phenogroups appear more significant on the Y axis (-log10(P-value)). Candidate genes are annotated on the plot. The red line depicts the genome-wide significant line (p = 5 x 10-8).

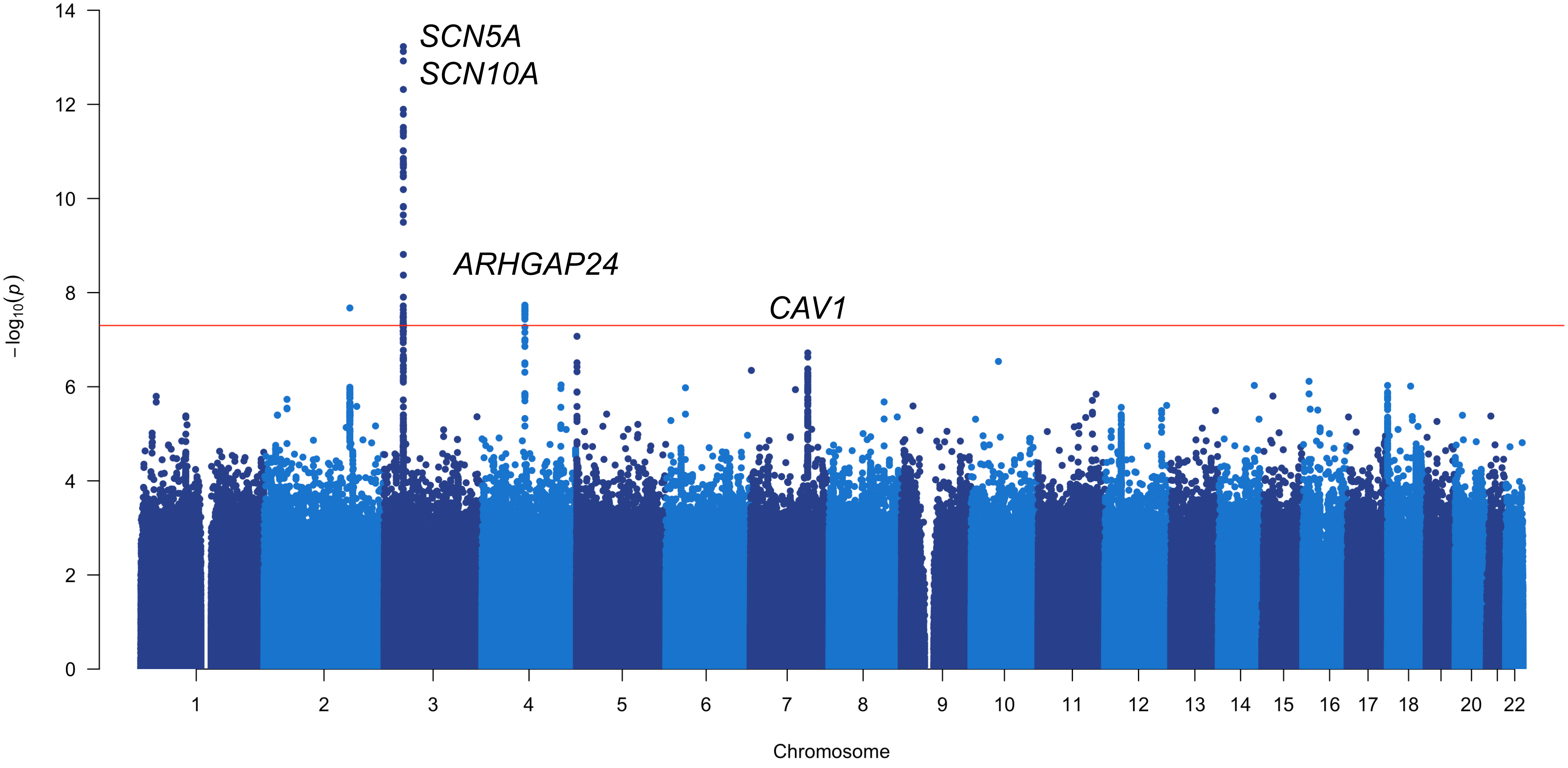

Supplement: Supplementary file 1 [file hcq-17-e010602-s001.pdf]
