# Supplementary material for: Prognostic Significance and Associations of Neural Network–Derived Electrocardiographic Features
Source: Circ Cardiovasc Qual Outcomes. 2024 Nov 14;17(12):e010602. doi: 10.1161/CIRCOUTCOMES.123.010602 (PMC7616866; doi:10.1161/CIRCOUTCOMES.123.010602)
Supplement: Supplementary file 3 [file hcq-17-e010602-s003.pdf]

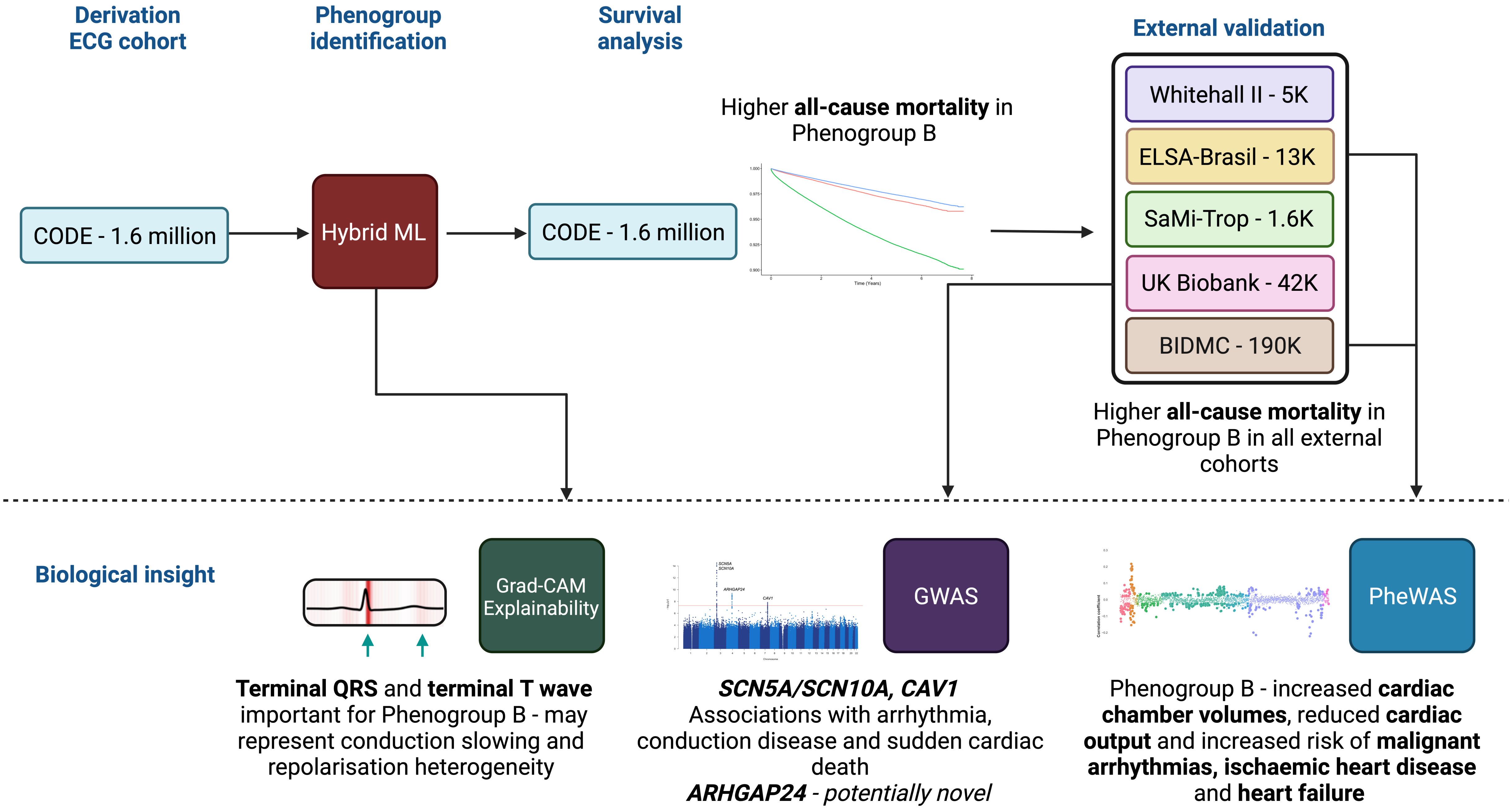

Derivation ECG cohort
Phenogroup identification
Survival analysis
External validation
CODE - 1.6 million
Hybrid ML
CODE - 1.6 million
Higher **all-cause mortality** in Phenogroup B
Time (Years)
Whitehall II - 5K
ELSA-Brasil - 13K
SaMi-Trop - 1.6K
UK Biobank - 42K
BIDMC - 190K
Higher **all-cause mortality** in Phenogroup B in all external cohorts
Biological insight
Grad-CAM Explainability
GWAS
PheWAS
Terminal QRS and **terminal T wave** important for Phenogroup B - may represent conduction slowing and repolarisation heterogeneity
SCN5A/SCN10A, CAV1
Associations with arrhythmia, conduction disease and sudden cardiac death
ARHGAP24 *- potentially novel*
Phenogroup B - increased **cardiac chamber volumes**, reduced **cardiac output** and increased risk of **malignant arrhythmias, ischaemic heart disease** and **heart failure**
